# Comparison of aldehyde-producing activities of cyanobacterial acyl-(acyl carrier protein) reductases

**DOI:** 10.1186/s13068-016-0644-5

**Published:** 2016-11-01

**Authors:** Hisashi Kudo, Ryota Nawa, Yuuki Hayashi, Munehito Arai

**Affiliations:** 1 Department of Life Sciences, Graduate School of Arts and Sciences, The University of Tokyo, 3-8-1 Komaba, Meguro, Tokyo 153-8902 Japan; 2 Department of Pure and Applied Sciences, College of Arts and Sciences, The University of Tokyo, 3-8-1 Komaba, Meguro, Tokyo 153-8902 Japan; 3 Precursory Research for Embryonic Science and Technology, Japan Science and Technology Agency, 4-1-8 Honcho, Kawaguchi, Saitama 332-0012 Japan

**Keywords:** Acyl-ACP reductase, Cyanobacteria, Fatty aldehyde, Aldehyde-deformylating oxygenase, Bioalkane

## Abstract

**Background:**

Biosynthesis of alkanes is an attractive way of producing substitutes for petroleum-based alkanes. Acyl-[acyl carrier protein (ACP)] reductase (AAR) is a key enzyme for alkane biosynthesis in cyanobacteria and catalyzes the reduction of fatty acyl-ACP to fatty aldehydes, which are then converted into alkanes/alkenes by aldehyde-deformylating oxygenase (ADO). The amino acid sequences of AARs vary among cyanobacteria. However, their differences in catalytic activity, substrate specificity, and solubility are poorly understood.

**Results:**

We compared the aldehyde-producing activity, substrate specificity, and solubility of AARs from 12 representative cyanobacteria. The activity is the highest for AAR from *Synechococcus elongatus* PCC 7942, followed by AAR from *Prochlorococcus marinus* MIT 9313. On the other hand, protein solubility is high for AARs from PCC 7942, *Microcystis aeruginosa*, *Thermosynechococcus elongatus* BP-1, *Synechococcus* sp. RS9917, and *Synechococcus* sp. CB0205. As a consequence, the amount of alkanes/alkenes produced in *Escherichia coli* coexpressing AAR and ADO is the highest for AAR from PCC 7942, followed by AARs from BP-1 and MIT 9313. Strikingly, AARs from marine and freshwater cyanobacteria tend to have higher specificity toward the substrates with 16 and 18 carbons in the fatty acyl chain, respectively, suggesting that the substrate specificity of AARs correlates with the type of habitat of host cyanobacteria. Furthermore, mutational analysis identified several residues responsible for the high activity of AAR.

**Conclusions:**

We found that the activity, substrate specificity, and solubility are diverse among various AARs. Our results provide a basis for selecting an AAR sequence suitable for metabolic engineering of bioalkane production while regulating carbon chain length.

**Electronic supplementary material:**

The online version of this article (doi:10.1186/s13068-016-0644-5) contains supplementary material, which is available to authorized users.

## Background

Biosynthesis of alkanes is an attractive way of producing substitutes for petroleum-based alkanes. Cyanobacteria are known to produce alkanes of 15–19 carbons in length [1, 2] and have attracted attention as a promising microbial cell factory for diesel fuels [3, 4]. The cyanobacterial alkane synthesis pathway involves two proteins, acyl-(acyl carrier protein (ACP)) reductase (AAR) and aldehyde-deformylating oxygenase (ADO) [5]. AAR catalyzes the reduction of fatty acyl-ACP or fatty acyl-CoA (which is an intermediate of fatty acid metabolism) into fatty aldehydes using NADPH [5]. Subsequently, ADO converts fatty aldehydes into alkanes (or alkenes if the fatty acyl group in fatty acyl-ACP/CoA is unsaturated) [5, 6]. Heterologous coexpression of cyanobacterial AAR and ADO in *Escherichia coli* results in production and secretion of alkanes [5]. Therefore, AAR and ADO are the key enzymes for biosynthesis of alkanes.

Genome analysis has shown that 90 % of cyanobacteria have both the *AAR* and *ADO* genes [7]. Moreover, the amino acid sequences of AAR vary among cyanobacteria, with an average sequence identity of ~67 % (Additional file 1: Table S1). To date, AAR and ADO from various cyanobacterial strains, including *Synechococcus elongatus* PCC 7942, *Nostoc punctiforme* PCC 73102, *Synechocystis* sp. PCC 6803, and *Prochlorococcus marinus* MIT 9313, have been used in metabolic engineering of biofuel production [5, 8–18]. Schirmer et al. reported that ADO from *Nostoc punctiforme* PCC 73102 has the highest alkane-producing activity among the ADOs from eight selected cyanobacterial strains [5]. It is still unknown, however, which cyanobacterial strains have AARs with higher aldehyde-producing activity. Finding a highly active AAR will contribute to improving the efficiency of bioalkane production in cyanobacteria, *E. coli*, and other organisms.

Here, we compared the aldehyde-producing activity, substrate specificity, and solubility of AARs from 12 representative cyanobacterial strains. We found that AAR from *Synechococcus elongatus* PCC 7942 has the highest activity and solubility. In addition, we found differences in substrate specificity between AARs from marine and freshwater cyanobacteria. Moreover, mutational analysis identified several residues responsible for the high activity of AAR. The results provide a basis for selecting an AAR sequence suitable for metabolic engineering of bioalkane production while regulating carbon chain length.

## Results

### Selection of representative AARs

We selected representative AAR sequences for activity measurements on the basis of the following two criteria. First, AARs that have been previously used in alkane biosynthesis studies, including AARs from *Synechococcus elongatus* PCC 7942 (7942 AAR), *Nostoc punctiforme* PCC 73102 (73102 AAR), S*ynechocystis* sp. PCC 6803 (6803 AAR), and *Prochlorococcus marinus* MIT 9313 (9313 AAR) [5, 8–18], are considered representative and were used in the present study.

**Fig. 1:**
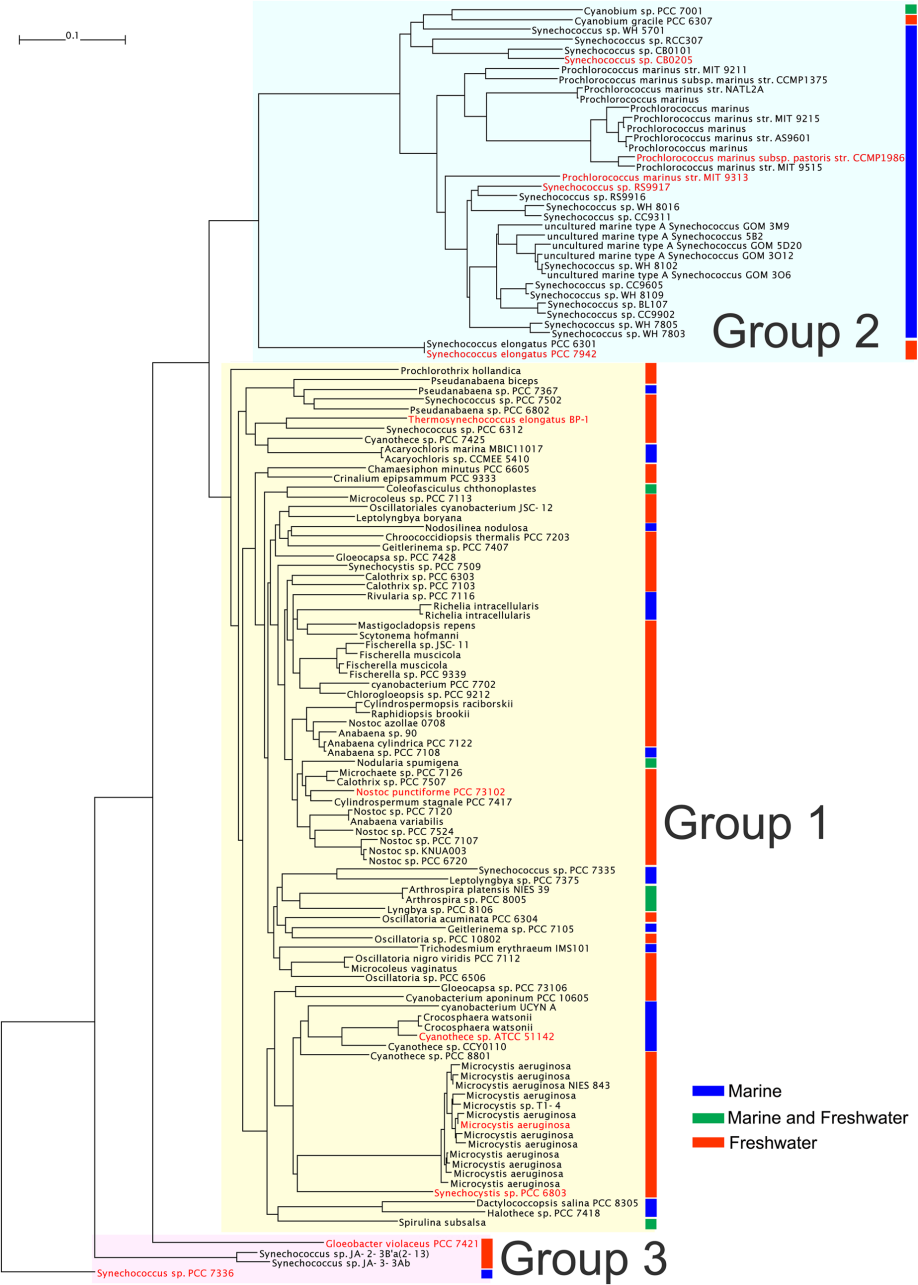
A phylogenetic tree of cyanobacteria on the basis of AAR amino acid sequences. Groups 1, 2, and 3 of cyanobacterial strains are hatched in *yellow*, *cyan*, and *magenta*, respectively. Host strains of the AARs used in this study are written in *red*. *Vertical bars* show habitats of cyanobacteria: in marine environments (*blue*), both in marine and freshwater environments (*green*), and in freshwater environments (*red*). The scale of the phylogenetic tree is shown in the *upper left corner*

Second, typical AAR sequences were selected from a phylogenetic tree of cyanobacteria constructed on the basis of the AAR amino acid sequences (Fig. 1). The phylogenetic tree showed that there are two large groups (Groups 1 and 2) and one small group (Group 3) of cyanobacterial strains (Fig. 1). Group 1 contains 88 strains, which are mainly freshwater cyanobacteria, including *Synechocystis* sp. PCC 6803 and *Nostoc punctiforme* PCC 73102. This group also contains a cluster of strains related to *Microcystis aeruginosa*, and thus, AAR from *Microcystis aeruginosa* (*Ma* AAR) was used in the present study. In addition, AAR from *Thermosynechococcus elongatus* BP-1 (BP-1 AAR) was selected because the AAR from a thermophilic cyanobacterium may have unique properties.

Group 2 contains 36 strains, which are mainly marine cyanobacteria, including *Prochlorococcus marinus* MIT 9313. This group also contains *Synechococcus elongatus* PCC 7942, which is a freshwater cyanobacterium, suggesting that 7942 AAR may have features in between those of AARs from freshwater and marine cyanobacteria.

On the other hand, Group 3 contains only four strains, but their AAR sequences are distinct from those categorized into Groups 1 and 2. Therefore, two of them, i.e., AARs from *Synechococcus* sp. PCC 7336 (7336 AAR) and *Gloeobacter violaceus* PCC 7421 (7421 AAR), which are marine and freshwater cyanobacteria, respectively, were selected and used in this study.

**Fig. 2:**
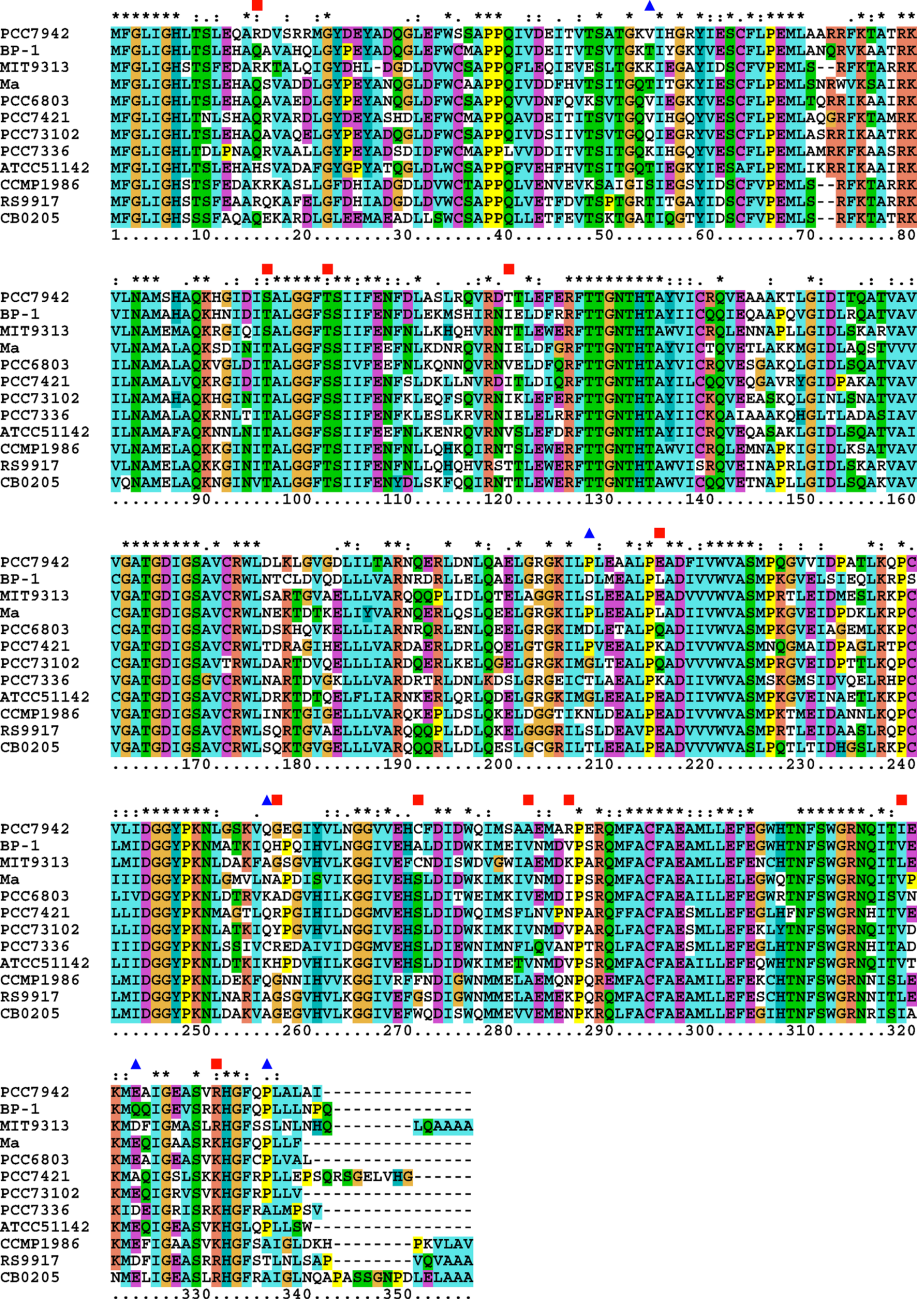
Amino acid sequences of representative AARs. The *asterisk* denotes fully conserved residues, and *colon* and *dot* denote partially conserved residues. *Red squares* and *blue triangles* show the residues that may be responsible for the activity and substrate specificity of AARs, respectively

Thus, a total of eight representative AAR sequences were selected for activity assays (Fig. 2). Sequence identities among them are 53–76 % (Additional file 2: Table S2).

### AAR activity

It is known that AAR proteins are prone to aggregation, and the 73102 AAR protein forms inclusion bodies when overexpressed in *E. coli* [19]; this situation precludes in vitro characterization of the catalytic activity of AAR. Therefore, we initially studied the amount of aldehydes produced by AAR in *E. coli* cell cultures. Nonetheless, aldehydes are unstable in *E. coli* and are converted into alcohols by endogenous enzymes in *E. coli* [5]. In fact, gas chromatography-mass spectrometry (GC–MS) analyses of *E. coli* cell cultures expressing the 7942 AAR protein could not detect aldehydes (Additional file 3: Figure S1a). Thus, to convert aldehydes into alkanes/alkenes, which are more stable than aldehydes, we coexpressed ADO from *Nostoc punctiforme* PCC 73102 (73102 ADO) with AAR in *E. coli*. A GC–MS profile of the *E. coli* cell culture coexpressing AAR and ADO showed predominant amounts of heptadecene and pentadecane and a small amount of heptadecane (Additional file 3: Figure S1b–d) [14]. The absolute amount of hydrocarbons produced in *E. coli* coexpressing 7942 AAR and 73102 ADO was 12 mg/L culture. Control experiments showed that hydrocarbons were produced in *E. coli* only when both AAR and ADO were coexpressed (Additional file 3: Figure S1a).

**Fig. 3:**
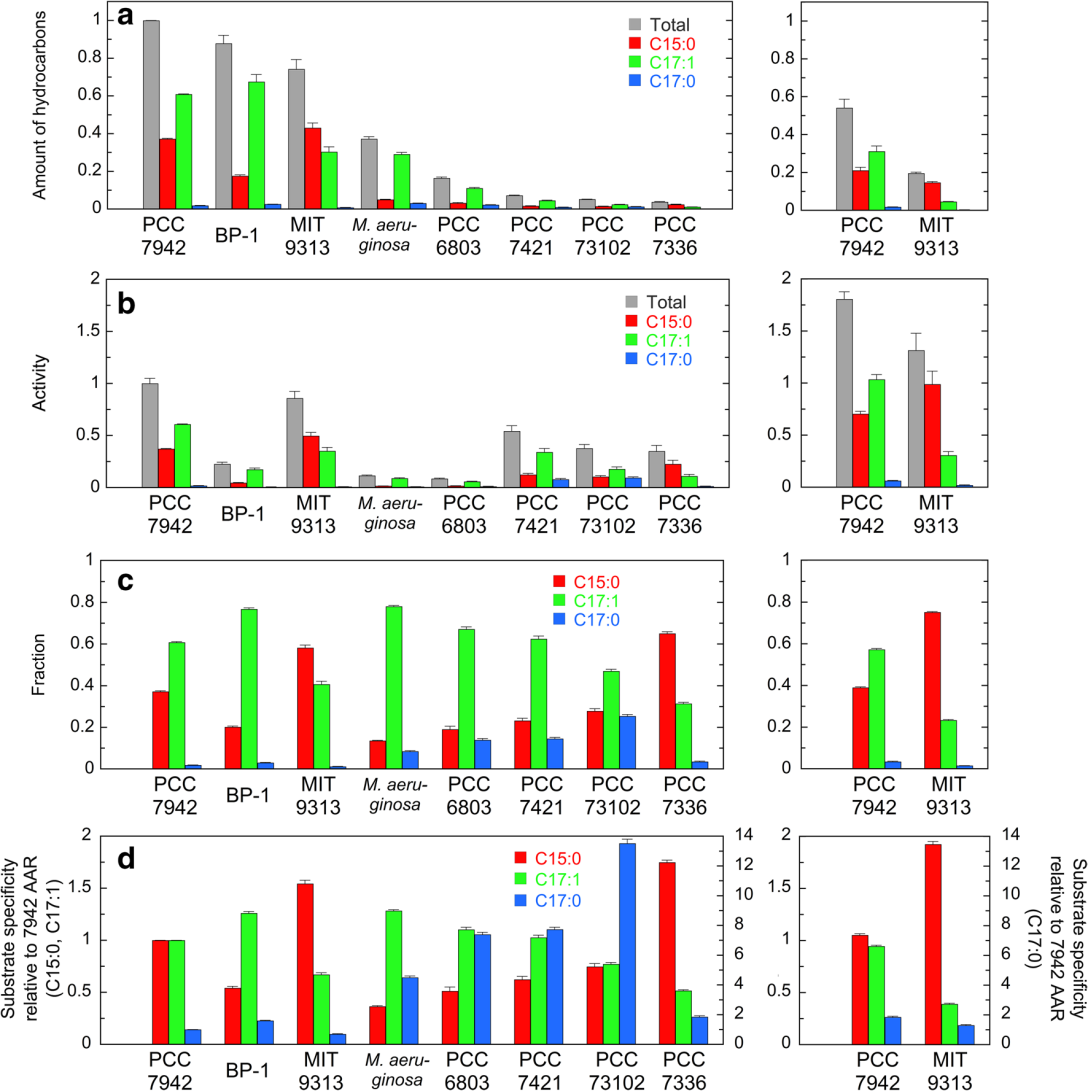
Activity and substrate specificity of eight representative AARs. **a** The amounts of hydrocarbons produced in *E. coli* coexpressing AAR and 73102 ADO. The amounts of pentadecane (C15:0), heptadecene (C17:1), and heptadecane (C17:0) and their combined total amount are shown in* red, green, blue, and gray*, respectively. The values are normalized to the total amount of hydrocarbons produced in *E. coli* coexpressing 7942 AAR and 73102 ADO. **b** The aldehyde-producing activity of AAR relative to that of 7942 AAR. The values are expressed as the amount of hydrocarbons normalized to the amount of the soluble form of AAR. **c** Fractions of pentadecane, heptadecene, and heptadecane relative to the total amount of hydrocarbons produced in *E. coli*, indicating the substrate specificity of AAR. **d** The substrate specificity of AAR relative to that of 7942 AAR. The *vertical axis* for pentadecane and heptadecene is shown on the *left* side, and that for heptadecane is shown on the *right* side. In all panels, the abscissa shows the names of host strains of AARs, and the *right* graph indicates the results for 7942 AAR and 9313 AAR with plasmids containing a mutant T7 promoter; in such cases, the expression level of AAR was decreased

Figure 3a shows the amounts of pentadecane, heptadecene, and heptadecane and their total amount in *E. coli* cell cultures coexpressing AAR from one of eight representative cyanobacteria and 73102 ADO. The total amount of hydrocarbons produced in *E. coli* is the highest for 7942 AAR, followed by BP-1 AAR, 9313 AAR, *Ma* AAR, 6803 AAR, 7421 AAR, 73102 AAR, and 7336 AAR in decreasing order. Those for 7421 AAR, 73102 AAR, and 7336 AAR are less than 10 % as compared with 7942 AAR.

**Fig. 4:**
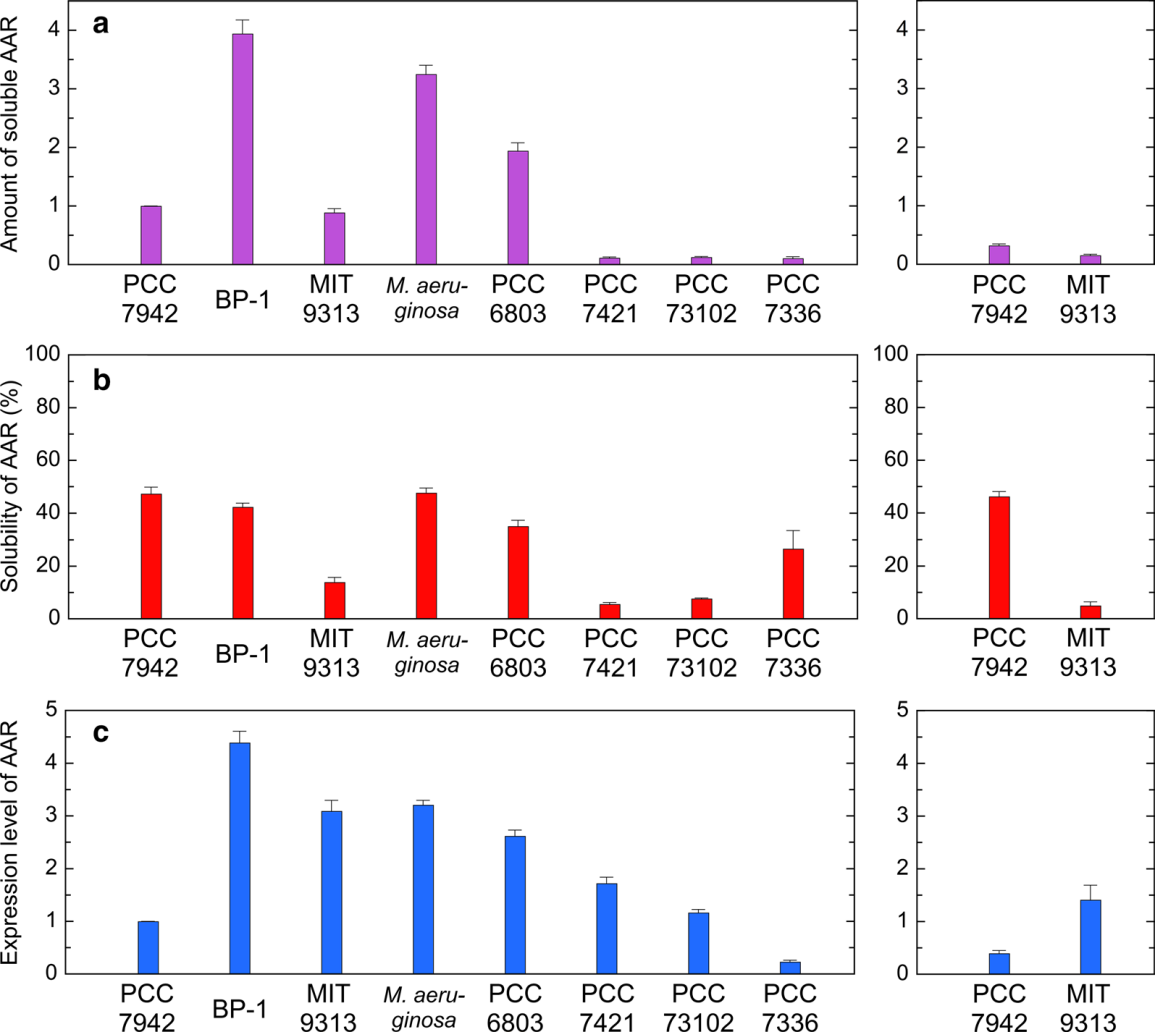
The solubility and expression level of eight representative AARs quantified by western blotting. **a** The amount of the soluble form of AAR. **b** Solubility (%) of AAR calculated as the ratio of the amount of the soluble form to the total amount of the soluble and insoluble forms of AAR. **c** The expression level of AAR, calculated as the total amount of the soluble and insoluble forms, is normalized to that of 7942 AAR

**Fig. 5:**
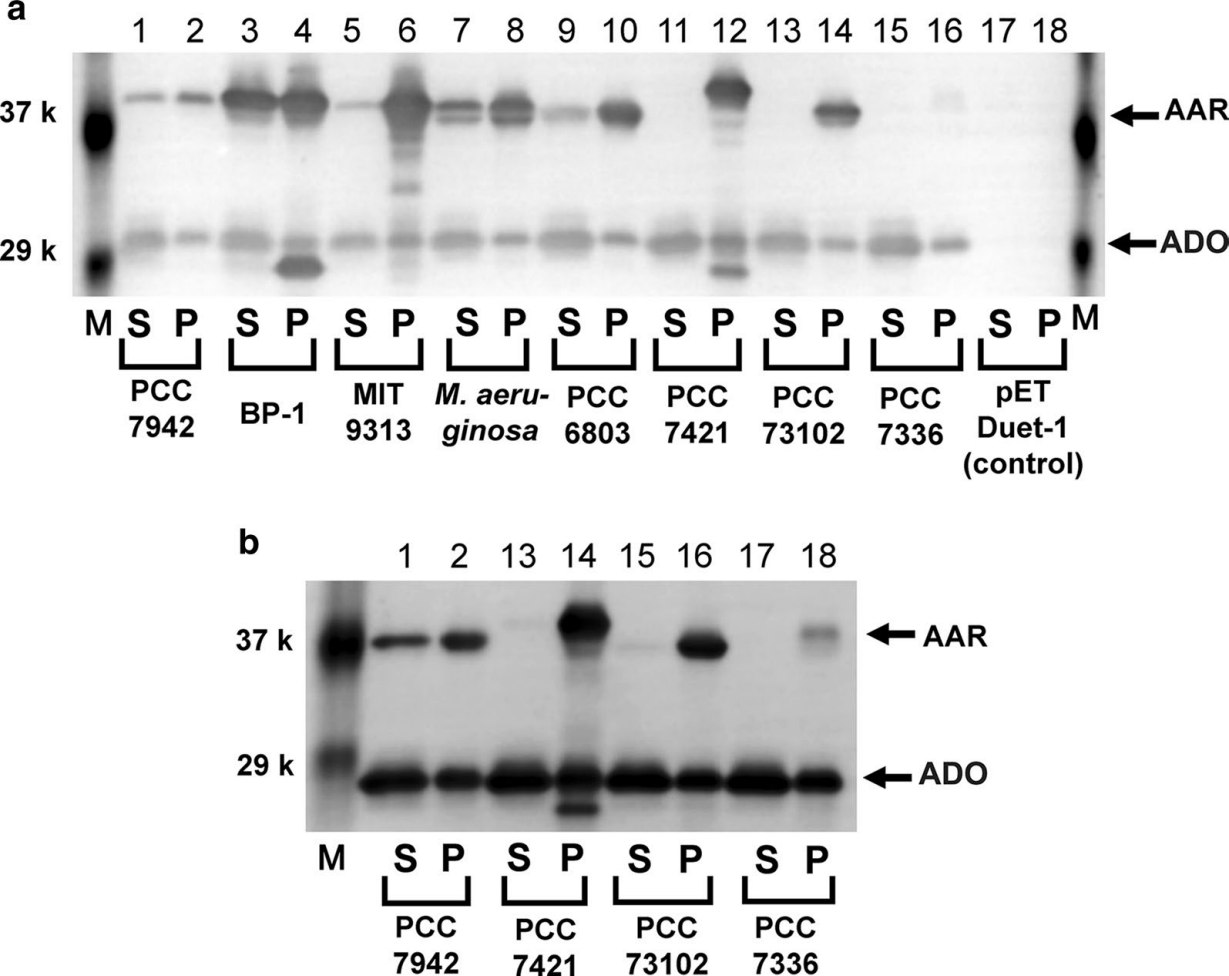
Western blotting of the supernatant and pellet fractions of the *E. coli* cell culture lysates. The *E. coli* cell culture coexpressing AAR and 73102 ADO was sonicated and centrifuged to separate the supernatant (*S*) and pellet (*P*) fractions. *M* denotes the molecular weight markers. The bands for AAR (38.6–39.8 kDa) and ADO (27.4 kDa) are indicated by *arrows*. The results for *E. coli* transformed with the pETDuet-1 vector containing neither the *AAR* nor *ADO* gene are shown in *lanes* 17 and 18 as a control. In **a** the coloring reaction lasted for ~2 min, while in **b** the reaction lasted more than 10 min for detection of low amounts of the soluble form of 7421 AAR, 73102 AAR, and 7336 AAR

Factors affecting the amount of hydrocarbons produced in *E. coli* are both the catalytic activity of AAR and the amount of the soluble form of AAR. Therefore, the total amount of hydrocarbons in the *E. coli* cell culture, normalized to the amount of the soluble form of AAR, was used as an index of the aldehyde-producing activity of AAR. Thus, the amount of the soluble form of AAR was quantified by western blotting (Figs. 4a, 5). There were large differences in the amount of the soluble form among various AARs. We found that the amount of the soluble protein is high for BP-1 AAR, *Ma* AAR, and 6803 AAR but low for 7421 AAR, 73102 AAR, and 7336 AAR (Figs. 4a, 5) . Note that the amount of the soluble form of ADO was the same for all cell cultures expressing various AAR proteins (Fig. 5), indicating that the solubility and the expression level of the ADO protein did not affect the amount of hydrocarbons produced in *E. coli*.

Figure 3b shows the relative aldehyde-producing activity of AAR expressed as the total amount of hydrocarbons in the *E. coli* cell culture (Fig. 3a) divided by the amount of the soluble form of AAR (Fig. 4a). The aldehyde-producing activity is the highest for 7942 AAR, followed by 9313 AAR, 7421 AAR, 73102 AAR, 7336 AAR, BP-1 AAR, *Ma* AAR, and 6803 AAR in decreasing order. Thus, although the amount of the soluble protein is high for BP-1 AAR, *Ma* AAR, and 6803 AAR, their catalytic activity is low.

When AAR is highly active, and the conversion of aldehydes into alkanes/alkenes by ADO is the rate-limiting step in alkane biosynthesis in *E. coli*, the amount of produced hydrocarbons is not proportional to the amount of aldehydes produced by AAR. Thus, an AAR activity, particularly that of the highly active 7942 AAR, for which the total amount of hydrocarbons is the highest, would not be correctly measured in the present in vivo activity assay using *E. coli* coexpressing AAR and ADO. Accordingly, to decrease the expression level of 7942 AAR, we introduced single mutations into the T7 promoter region upstream of the *AAR* gene in the expression vector. Promoter mutations were not introduced into the T7 promoter for the *ADO* gene. Six mutant plasmids were constructed according to a study investigating the effects of various single-base pair substitutions in the T7 promoter region on protein expression levels [20]. We found that for four out of six mutant plasmids, the amounts of produced hydrocarbons and the soluble form of AAR were less than 70 and 50 % compared with those of the wild-type T7 promoter, respectively (Additional file 4: Figure S2a,b). Moreover, the amount of produced hydrocarbons was proportional to the amount of the soluble form of AAR, indicating that hydrocarbon production was rate-limited by AAR activity. Consequently, the activity of 7942 AAR, obtained by averaging the activity measured using these four mutant plasmids (Fig. 3b; Additional file 4: Figure S2c), is ~1.8-fold greater than the activity of 7942 AAR measured using the wild-type T7 promoter. Because the amount of the soluble form of ADO was almost unaffected by the mutations (Additional file 4: Figure S2e), the observed changes in the amount of produced hydrocarbons could be attributed to the changes in the amount of the soluble form of AAR.

We also performed the same experiments for other AARs using a weakened T7 promoter. Here, we introduced a -13A>G mutation into the T7 promoter (i.e., adenine at the -13th position in the T7 promoter region was replaced by guanine) upstream of the *AAR* gene in each plasmid for the expression of other AARs. The promoter mutation resulted in the reduction of both the amount of soluble AAR and the production of hydrocarbons, indicating that hydrocarbon production was rate-limited by the AAR activity (Figs. 3a and 4a; Additional file 5: Figure S3a,b). Even when the mutation was introduced, the trends for both the activity and the amount of hydrocarbons are essentially the same as those observed without the promoter mutation, although the expression of 73102 AAR and 7336 AAR, which was already low without the promoter mutation, was undetectable when the promoter mutation was introduced. We found that the activity of 9313 AAR was ~1.4-fold greater than the 7942 AAR activity measured using the wild-type T7 promoter (Additional file 5: Figure S3c). Because the activity of 9313 AAR measured using the wild-type T7 promoter was close to that of 7942 AAR (Fig. 3b), it was possible that the conversion of aldehydes into alkanes/alkenes by ADO is the rate-limiting step in alkane biosynthesis in *E. coli* harboring the wild-type T7 promoter. Thus, the activities of 7942 AAR and 9313 AAR are the highest and second highest, respectively.

### Solubility and expression level

The solubility and expression level of AAR in *E. coli* were quantified by western blotting. Here, the expression level was determined as the total amount of both the soluble and insoluble forms of AAR, while the solubility was estimated as the ratio of the amount of the soluble form to the total amount of the soluble and insoluble forms of AAR. We found that the solubility and the expression level are highly diverse among AARs (Figs. 4, 5). Although all AAR proteins studied here are less than 50 % soluble and are prone to aggregation, solubility is the highest for 7942 AAR and *Ma* AAR, followed by BP-1 AAR, 6803 AAR, and 7336 AAR (Fig. 4b). Remarkably, 9313 AAR, 73102 AAR, and 7421 AAR are only less than 20 % soluble, and more than 80 % of these proteins are precipitated inside *E. coli* cells. Nonetheless, the expression level of the 9313 AAR protein is significantly higher, and the amount of the soluble form of 9313 AAR, which corresponds to multiplication of the expression level and the solubility, reaches the same level as observed for 7942 AAR.

The high solubility of BP-1 AAR could be explained by the fact that proteins derived from thermophilic bacteria have high stability [21]. BP-1 AAR also showed the highest expression level (Fig. 4c), thereby yielding the highest amount of soluble protein (Fig. 4a) and high hydrocarbon productivity (Fig. 3a).

### Substrate specificity

There were differences in substrate specificity among AARs (Fig. 3c, d). For six out of the eight AARs used above, which are derived from freshwater cyanobacteria, the major product is heptadecene, indicating that these AARs have higher substrate affinity for 18-carbon fatty acyl-ACP/CoA as a substrate, because ADO removes the CO moieties of aldehydes. This is particularly remarkable for *Ma* AAR, BP-1 AAR, and 6803 AAR; the amount of pentadecane produced is only 20 % that of heptadecene. The results are consistent with the observation that *Synechocystis* sp. PCC 6803 produces heptadecane but not pentadecane [2, 5]. The difference in the primary product between *Synechocystis* sp. PCC 6803 (heptadecane) and *E. coli* coexpressing 6803 AAR and 73102 ADO (heptadecene) may be due to the difference in the abundance of the substrates, stearoyl-ACP/CoA and oleoyl-ACP/CoA, in the cyanobacterium and *E. coli*. In fact, acyl-ACPs are saturated in cyanobacteria because desaturation of fatty groups occurs after fatty acids are synthesized and integrated into the thylakoid membrane [22]. On the other hand, most acyl-ACPs are desaturated during fatty acid synthesis in *E. coli* [23]. Consequently, the major product is alkane and alkene in cyanobacteria and *E. coli*, respectively, when AAR and ADO are coexpressed.

By contrast, the major product is pentadecane for 9313 AAR and 7336 AAR (Fig. 3c), which are derived from marine cyanobacteria, indicating their higher substrate affinity for 16-carbon fatty acyl-ACP/CoA, i.e., palmitoyl-ACP/CoA, than for 18-carbon fatty acyl-ACP/CoA. The fraction of pentadecane increased to 75 % of total hydrocarbons for 9313 AAR using a weakened T7 promoter compared with that harboring the wild-type T7 promoter (Fig. 3c; Additional file 5: Figure S3d). Although the major product is heptadecene for 7942 AAR, pentadecane is also highly accumulated at ~40 % of the total hydrocarbons produced in *E. coli* (Fig. 3c; Additional file 4: Figure S2d).

Among the three types of hydrocarbons detected in *E. coli* coexpressing AAR and ADO, heptadecane exhibited the lowest production. Nevertheless, coexpression of 73102 AAR and 73102 ADO in *E. coli* resulted in high production of heptadecane at ~25 % of total hydrocarbons in *E. coli* (Fig. 3c).

**Fig. 6:**
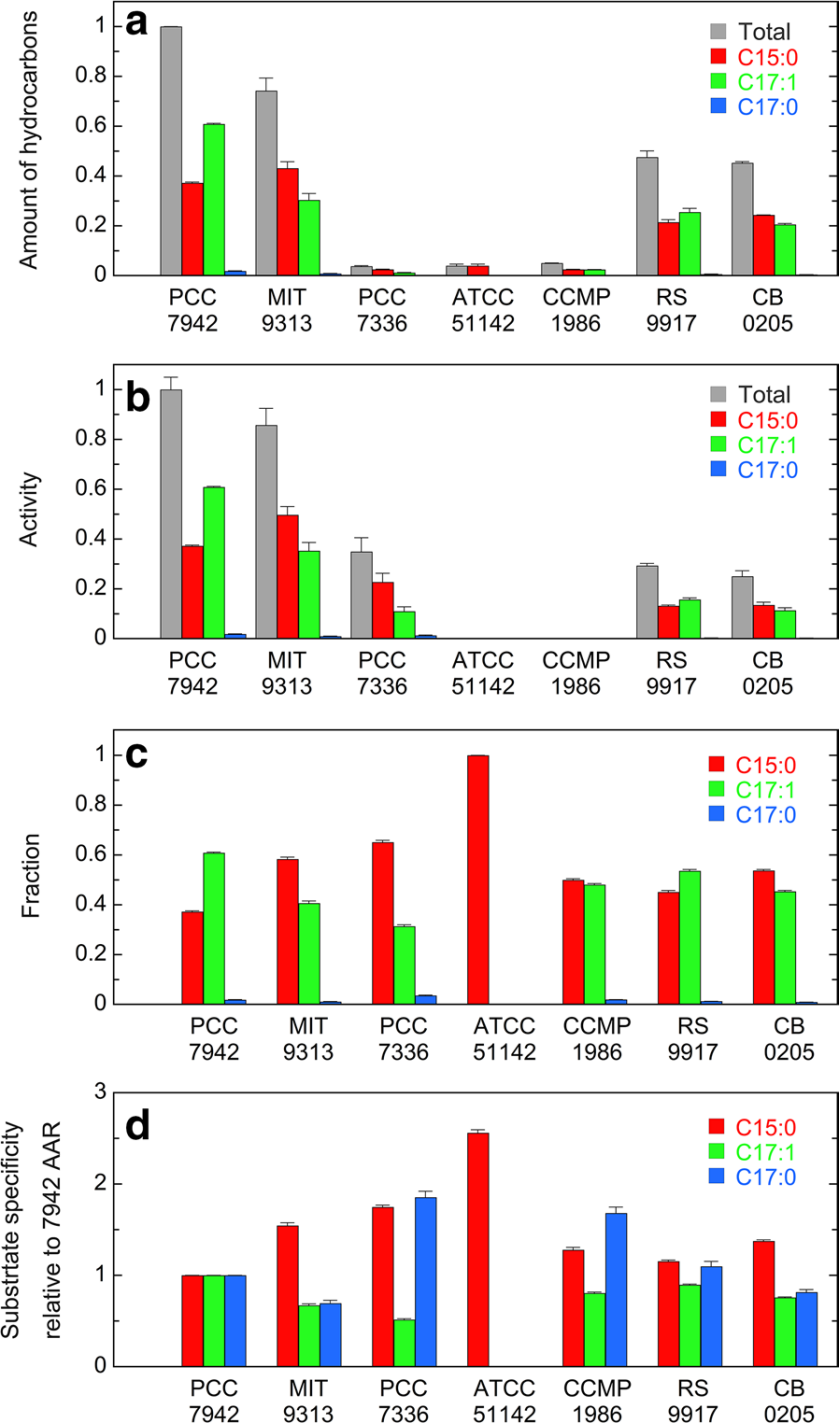
Activity and substrate specificity of AARs from marine cyanobacteria. **a** The amounts of hydrocarbons produced in *E. coli* coexpressing AAR and 73102 ADO. **b** The aldehyde-producing activity of AAR relative to that of 7942 AAR. The activities of 51142 AAR and 1986 AAR were not determined, because the amounts of the soluble form of 51142 AAR and 1986 AAR are too low to detect by western blotting (see Fig. 7). **c** Fractions of pentadecane, heptadecene, and heptadecane relative to the total amount of hydrocarbons produced in *E. coli*, indicating the substrate specificity of AAR. **d** The substrate specificity of AAR relative to that of 7942 AAR. The details are the same as shown in Fig. 3. The data for 7942 AAR are shown for comparison

**Fig. 7:**
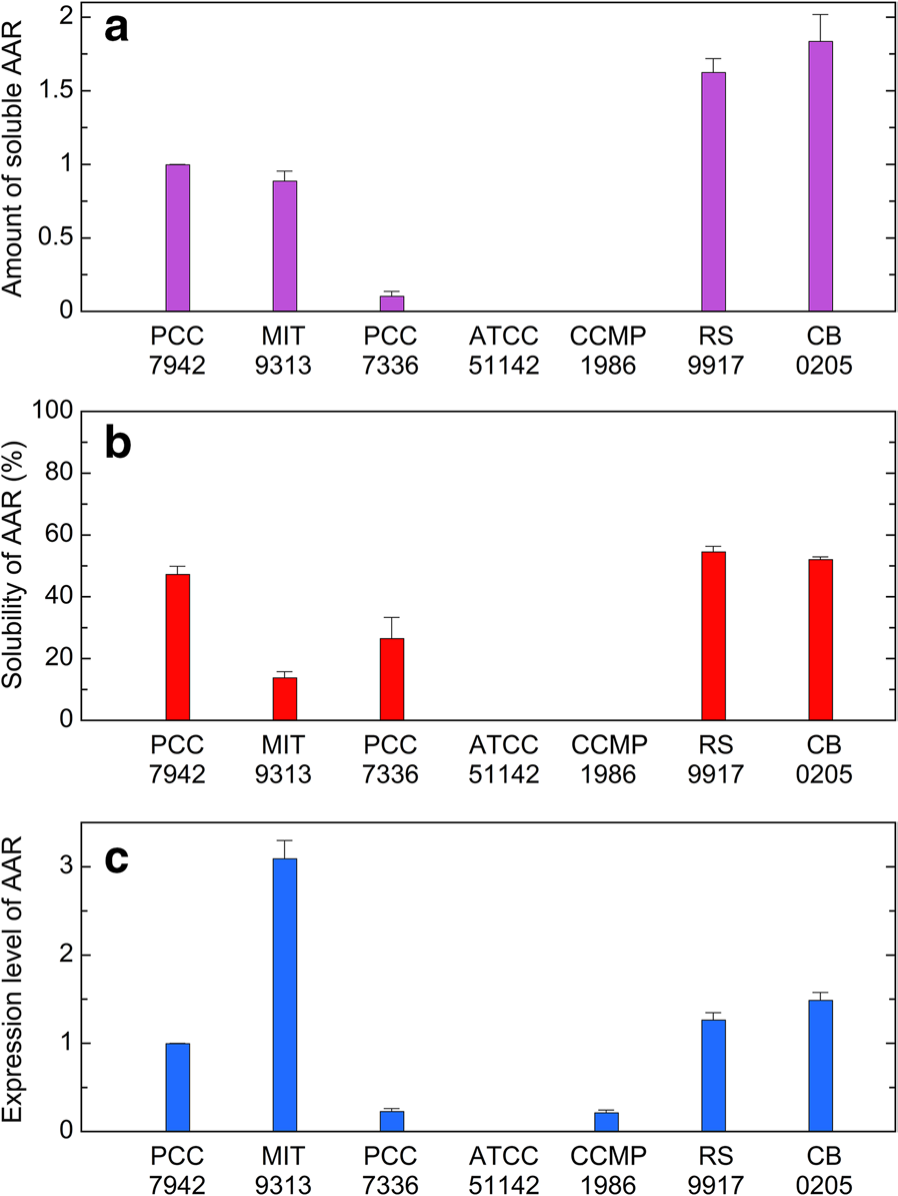
The solubility and expression level of AARs from marine cyanobacteria. **a** The amount of the soluble form of AAR. **b** Solubility (%) of AAR. **c** The expression level of AAR. The details are the same as shown in Fig. 4. The data for 7942 AAR are shown for comparison

Both 9313 AAR and 7336 AAR are derived from marine cyanobacteria, but other six AARs are from freshwater cyanobacteria. To increase the number of AARs derived from marine cyanobacteria, we also investigated the activity, substrate specificity, and solubility of four AARs from marine cyanobacteria, i.e., AARs from *Cyanothece* sp. ATCC 51142 (51142 AAR), *Prochlorococcus marinus* subsp. pastoris str. CCMP1986 (1986 AAR), *Synechococcus* sp. RS9917 (9917 AAR), and *Synechococcus* sp. CB0205 (0205 AAR). In the phylogenetic tree of AAR (Fig. 1), only 51142 AAR is included in Group 1 (mainly freshwater cyanobacteria), whereas the other AARs are included in Group 2 (mainly marine cyanobacteria). Notably, 51142 AAR, 1986 AAR, and 9917 AAR were previously used in alkane biosynthesis studies [5, 16]. Sequence identities among the 12 AARs used in this study are 53–82 % (Additional file 2: Table S2). The results show that the additional four AARs from marine cyanobacteria have higher substrate affinity for 16-carbon fatty acyl-ACP/CoA compared with AARs from freshwater cyanobacteria (Figs. 3d, 6). In particular, only pentadecane is produced for 51142 AAR, although its hydrocarbon productivity is low. The solubilities of 9917 AAR and 0205 AAR are as high as those of 7942 AAR and *Ma* AAR, whereas the amounts of the soluble form of 51142 AAR and 1986 AAR are too low to detect by western blotting (Fig. 7).

### Sequence determinants of AAR properties

To understand the sequence determinants of the catalytic activity, substrate specificity, and solubility of AARs, we carried out a correlation analysis between these properties and the amino acid sequences. Additional file 6: Figure S4a shows the correlation between an aldehyde-producing activity of an AAR and its sequence identity with the 7942 AAR sequence (Additional file 6: Figure S4a). The 7942 AAR sequence is used as a reference because this protein exhibited the highest activity. There appears to be a positive correlation, but when the data point for 7942 AAR is omitted, the correlation disappears (Additional file 6: Figure S4a), indicating that the activity level of AAR is not determined by overall similarity of amino acid sequences. Similarly, the substrate specificity of AAR, expressed as the fraction of pentadecane and heptadecane, does not correlate well with the sequence identity to the 9313 AAR and 73102 AAR sequences, respectively (Additional file 6: Figure S4b, d). Moreover, although both 9313 AAR and 7336 AAR have higher substrate specificity for palmitoyl-ACP/CoA, the sequence identity between them is only 53 % (Additional file 2: Table S2). Taken together, these results suggest that local differences in the amino acid sequences determine both the activity level of AAR and the substrate specificity for palmitoyl-ACP/CoA.

Similar to the activity of AAR, the solubility and expression level of AAR did not correlate well with the sequence similarity of AARs (Additional file 6: Figure S4e,f), suggesting that local differences in amino acid sequences determine the solubility of AARs. By contrast, the fraction of heptadecene weakly correlated with the sequence identity to the *Ma* AAR sequence (Additional file 6: Figure S4c). This finding suggests that AARs having an amino acid sequence similar to that of *Ma* AAR can produce a larger amount of heptadecene.

**Fig. 8:**
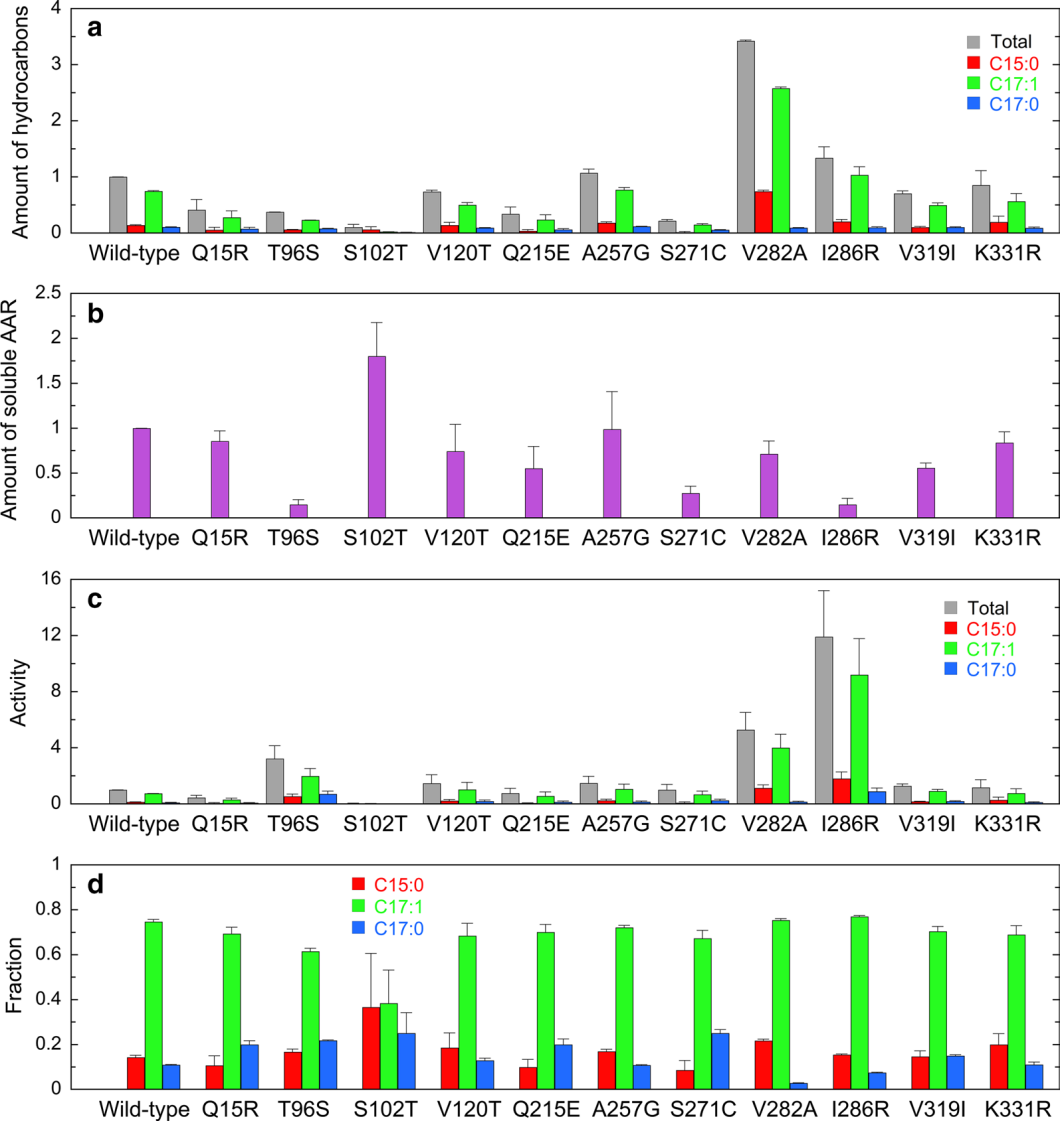
Activity and substrate specificity of mutants of 6803 AAR. **a** The amounts of hydrocarbons produced in *E. coli* coexpressing mutants of 6803 AAR and 73102 ADO. **b** The amount of the soluble form of a 6803 AAR mutant. **c** The aldehyde-producing activity of a 6803 AAR mutant relative to that of the wild-type 6803 AAR. **d** Fractions of pentadecane, heptadecene, and heptadecane relative to the total amount of produced hydrocarbons, indicating the substrate specificity. The details are the same as shown in Figs. 3, 4

To search for the amino acid residues responsible for high activity and high specificity for palmitoyl-ACP/CoA, we carefully analyzed the multiple sequence alignment of AARs (Fig. 2). First, we searched for the residues that are conserved for highly active AARs, i.e., 7942 AAR and 9313 AAR, but are not conserved for other AARs. The following 11 residues match these criteria: Arg15, Ser96, Thr102, Thr120, Glu215, Gly257, Cys271, Ala282, Arg286, Ile319, and Arg331 (in terms of the 7942 AAR sequence) (red squares in Fig. 2). We introduced single amino acid substitutions into 6803 AAR, which has low activity, at one of the above 11 residues conserved in highly active AARs. We constructed 11 mutants of 6803 AAR (Q15R, T96S, S102T, V120T, Q215E, A257G, S271C, V282A, I286R, V319I, and K331R) and investigated the effects of the mutations on the AAR activity (Fig. 8). As expected, the activities of T96S, V282A, and I286R mutants of 6803 AAR were increased by approximately 4-, 5-, and 12-fold, respectively, compared with that of the wild-type 6803 AAR. On the other hand, introduction of Q15R and S102T mutations decreased the activity of 6803 AAR, indicating that these residues are important for maintaining the activity of 6803 AAR.

**Fig. 9:**
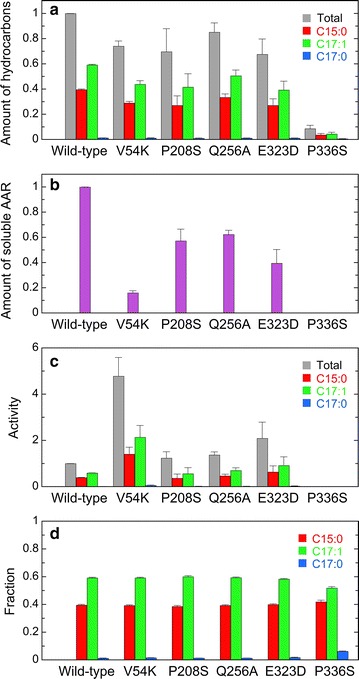
Activity and substrate specificity of mutants of 7942 AAR. **a** The amounts of hydrocarbons produced in *E. coli* coexpressing mutants of 7942 AAR and 73102 ADO. **b** The amount of the soluble form of a 7942 AAR mutant. **c** The aldehyde-producing activity of a 7942 AAR mutant relative to that of the wild-type 7942 AAR. The activity of the P336S mutant was not determined, because the amount of soluble AAR was too low to detect by western blotting. **d** Fractions of pentadecane, heptadecene, and heptadecane relative to the total amount of produced hydrocarbons, indicating the substrate specificity. The details are the same as shown in Figs. 3, 4

Second, we searched for the residues that are similar in 9313 AAR and 7336 AAR but are not in other AARs. There are at least five residues that match these criteria: Val54, Pro208, Gln256, Glu323, and Pro336 (in terms of the 7942 AAR sequence) (blue triangles in Fig. 2). We introduced single amino acid substitutions into 7942 AAR, which exhibited higher affinity for oleoyl-ACP/CoA as a substrate, with the residues similar to those in 9313 AAR and 7336 AAR and constructed five mutants of 7942 AAR (V54K, P208S, Q256A, E323D, and P336S). The results show that substrate specificity of the mutants was almost unchanged (Fig. 9). The fraction of pentadecane was slightly increased for the P336S mutant, but the total amount of hydrocarbons was decreased, because the amount of soluble AAR was very low. These results suggest that it is difficult to change the substrate specificity of AARs using single amino acid substitutions. Notably, the activity of the V54K mutant of 7942 AAR is 4.8-fold higher than that of the wild-type 7942 AAR. However, the amount of soluble AAR is only 0.16-fold that of the wild-type AAR, resulting in hydrocarbon production levels approximately 0.8-fold that of the wild-type AAR.

## Discussion

### Activity and solubility

In this study, we carefully examined the aldehyde-producing activity of various AARs by measuring both hydrocarbon production and the amount of soluble AAR. In general, normalization of the amount of a product to the amount of a soluble protein is necessary for determining the activity of an enzyme, when in vivo activity is measured using an organism expressing an enzyme. In fact, although hydrocarbon production is high for *E. coli* expressing BP-1 AAR (Fig. 3a), the amount of soluble AAR is also high (Fig. 4a), resulting in a low activity (Fig. 3b). On the other hand, the amounts of the product and soluble protein are low for 7421 AAR (Figs. 3a, 4a), although its activity is the third highest (Fig. 3b). Moreover, quantitative analysis of the soluble and insoluble forms of a protein reveals the solubility and the expression level of a protein.

Here, we clearly show that 7942 AAR has the highest activity and solubility. The results suggest that 7942 AAR should be used in metabolic engineering of bioalkane production. Recently, Coursolle et al. studied activities of several AARs, including 7942 AAR, by comparing the amount of alcohols, which are probably produced from aldehydes in *E. coli*, and suggested that 7942 AAR has the highest activity [16]. However, alcohol production was not normalized to the amount of the soluble form of AAR, and thus the exact comparison of the AAR activity was not carried out in their study. By contrast, here, we measured the amounts of both the hydrocarbons and the soluble forms of AAR and demonstrated that 7942 AAR has the highest activity and solubility.

We also found that although BP-1 AAR is highly soluble, its activity is low at 37 °C. In general, enzymes from thermophilic bacteria have high optimal temperatures and low activity at 25–37 °C due to the slower dynamics of the enzymes [24]. Thus, BP-1 AAR may be useful when aldehyde production is conducted at higher temperatures.

Recently, Warui et al. reported that aldehydes produced by AAR are efficiently delivered to ADO through the binding of AAR to ADO [18]. It is possible that AAR and ADO that are derived from the same strain interact with each other more efficiently than those derived from different strains, resulting in higher production of hydrocarbons. Here, we used *E. coli* coexpressing AAR from one of 12 representative cyanobacterial strains and 73102 ADO, and we found that the combination of AAR and ADO, both of which are derived from *Nostoc punctiforme* PCC 73102, does not show the highest activity but instead exhibited rather low activity (Fig. 3b). These results suggest that the binding site for ADO on AAR is conserved among the AARs used in the present study. An alternative explanation may be that the ADO-binding site on AAR is not conserved and that 73102 AAR binds to 73102 ADO most strongly, whereas other AARs have much higher activity than 73102 AAR. In both cases, it is clear that 7942 AAR and 9313 AAR have a high aldehyde-producing activity.

In the present mutational studies, we succeeded in identifying three residues that conferred AARs with high activity (i.e., Ser96, Ala282, and Arg286) (Fig. 8). Because Ala282 and Arg286 are not conserved residues (Fig. 2), mutations at these sites may increase the activity of both 6803 AAR and other less active AARs.

### Substrate specificity

Remarkably, there are differences in the chain length of products of various AARs. 9313 AAR, 7336 AAR, 51142 AAR, 1986 AAR, and 0205 AAR mainly produce hexadecanal, a precursor of pentadecane, and in addition, 9917 AAR produces more hexadecanal than 7942 AAR, indicating that these AARs have a higher affinity for palmitoyl-ACP/CoA as a substrate. On the other hand, other AARs mainly produce octadecenal, a precursor of heptadecene, indicating substrate specificity for 18-carbon fatty acyl-ACP/CoA. Our finding about the difference in the substrate specificity of AARs is useful for regulating the carbon chain length of aldehydes and hydrocarbons produced in cyanobacteria, *E. coli*, and other organisms using AAR and ADO.

Of particular interest is that AARs producing mainly hexadecanal are derived from marine cyanobacteria, but other AARs are from freshwater cyanobacteria. These results indicate that the substrate specificity of AAR depends on habitats of host strains, with AARs from marine and freshwater cyanobacteria having substrate specificity for 16- and 18-carbon fatty acyl-ACP/CoA, respectively. Moreover, the phylogenetic tree shows that many freshwater cyanobacteria are clustered in Group 1, which contains host strains of AARs that mainly produce octadecanal (Fig. 1). On the other hand, many marine cyanobacteria are clustered in Group 2, which includes host strains of AARs that mainly produce hexadecanal (Fig. 1). The clear separation of the habitats and substrate specificity in the phylogenetic tree suggests that the substrate specificity of AAR is conserved among closely related strains.

Although the habitat of *Synechococcus elongatus* PCC 7942 is freshwater, this cyanobacterium belongs to Group 2, which contains many marine cyanobacteria. Because 7942 AAR produces high amounts of 16- and 18-carbon aldehydes, these results suggest that 7942 AAR possesses characteristics in between those of AARs from marine and freshwater cyanobacteria.

Our present mutational studies suggest that single amino acid substitutions are not sufficient to change the substrate specificity of AARs (Fig. 9). In accordance with this, AARs having different substrate specificity are clustered into two separate groups in the phylogenetic tree. It is likely that many residues are responsible for determining the substrate specificity of AAR. However, each residue may only have small effects on substrate specificity. Combinations of multiple amino acid substitutions will be necessary for controlling the substrate specificity of AARs.

Recently, Shakeel et al. showed that marine cyanobacteria predominantly produce pentadecane, whereas freshwater cyanobacteria predominantly produce heptadecane [25]. Moreover, marine and freshwater cyanobacteria predominantly contain 16- and 18-carbon fatty acids, respectively, indicating that alkane chain length is primarily determined by fatty acid metabolism.

The substrate specificity of AARs from marine and freshwater cyanobacteria correlates well with the fatty acid composition in marine and freshwater cyanobacteria. Because long-chain fatty acids are synthesized through fatty acyl-ACP/CoA, the predominant production of 16- and 18-carbon fatty acids in marine and freshwater cyanobacteria indicates the presence of high amounts of 16- and 18-carbon acyl-ACP/CoA, respectively. Thus, the substrate specificity of AARs may be determined by the carbon chain length of the most abundant acyl-ACP/CoA in cyanobacteria. This situation may be due to evolutionary adaptation of AAR by shifting the substrate preference in response to changes in the habitat of the organism from marine to freshwater environment.

The reason why fatty acids and alkanes in marine cyanobacteria are shorter than those in freshwater cyanobacteria is unknown. One possible explanation may be that elongation of fatty acids and alkanes in cyanobacteria occurred when marine cyanobacteria expanded their habitats to freshwater environments, and accordingly, AAR from freshwater cyanobacteria appeared to be adapted to longer substrates. In line with this notion, there is an example of an evolutionary adaptation of substrate specificity in cyanobacterial enzymes, a lysophosphatidic acid acyltransferase (LPAAT), which provides fatty acids at the *sn-2* positions of glycerolipids [26]. The LPAAT from cyanobacteria is known to bind 16-carbon fatty acids, while the LPAAT localized at the endoplasmic reticulum in eukaryotic algae binds 18-carbon fatty acids, indicating that the substrate specificity of LPAAT changed during the evolution of plants from cyanobacteria to eukaryotic algae [26, 27].

## Conclusions

In this study, we selected 12 representative AARs and compared their aldehyde-producing activities. The results show that the activity, substrate specificity, solubility, and expression levels vary among AARs. The activity is high for 7942 AAR and 9313 AAR, while the solubility is high for 7942 AAR, *Ma* AAR, BP-1 AAR, 9917 AAR, and 0205 AAR. As a consequence, the amount of alkanes/alkenes produced in *E. coli* coexpressing AAR and ADO is the highest for 7942 AAR, followed by BP-1 AAR, 9313 AAR, and *Ma* AAR. Moreover, 9313 AAR, 7336 AAR, 51142 AAR, 1986 AAR, 9917 AAR, and 0205 AAR, derived from marine cyanobacteria, have higher substrate specificity for 16-carbon fatty acyl-ACP/CoA, while other AARs, derived from freshwater cyanobacteria, have higher specificity for 18-carbon fatty acyl-ACP/CoA, suggesting that substrate specificity of AARs correlates with the type of habitat of host cyanobacteria. Furthermore, mutational analysis identified several residues responsible for the high activity of AAR. Thus, the present results provide a basis for selecting an AAR sequence suitable for metabolic engineering of bioalkane production while regulating carbon chain length.

## Methods

### Plasmids

The DNA fragments encoding 6803 AAR, 73102 AAR, and 7942 AAR were prepared by overlap extension polymerase chain reaction. The codons were optimized for strong expression in *E. coli* [28]. The codon-optimized DNA fragments coding for BP-1 AAR, 7336 AAR, 7421 AAR, 9313 AAR, *Ma* AAR, 51142 AAR, 1986 AAR, 9917 AAR, and 0205 AAR were constructed by Eurofin Operon Biotechnologies. For coexpression of AAR and ADO in *E. coli*, the pETDuet-1 coexpression vector (Novagen) was used, into which the codon-optimized DNA fragments of AAR and ADO were cloned via the *Nco*I and *Eco*RI restriction sites and the *Nde*I and *Avr*II restriction sites, respectively [14]. Both the *AAR* and *ADO* genes are preceded by a T7 promoter, *lac* operator, and ribosome-binding site. 73102 ADO was used because it is known to have a high alkane-producing activity [5]. All the AAR and ADO proteins had a C-terminal extension of Gly–Ser–Ser–Gly and a 6× His-tag. All the AAR proteins had an additional Gly between the N terminus (Met) and the second residue in order to eliminate the frame shift due to the *Nco*I restriction site (CCATGG + GT). The ESPRESSO server, which estimates protein expression (http://cblab.meiyaku.jp/ESPRESSO/) [29], predicted that all the AAR and ADO constructs can be expressed in *E. coli*.

### Hydrocarbon production in *E. coli* coexpressing AAR and ADO

Alkanes/alkenes were produced in *E. coli* coexpressing AAR and ADO as described previously [14]. In brief, *E. coli* BL21(DE3)pLysS competent cells were transformed with the pETDuet-1 coexpression vector, containing both *AAR* and *ADO* genes, and inoculated onto a 2× YT agarose plate containing carbenicillin (50 μg/mL) and chloramphenicol (34 μg/mL). For control experiments, we used the pETDuet-1 vector containing only the 7942 *AAR* gene, the pET-15b vector containing only the 73102 *ADO* gene, and the pETDuet-1 vector containing neither the *AAR* nor *ADO* gene. The colonies on the plate were seeded into the M9 medium containing ampicillin (50 μg/mL) and chloramphenicol (34 μg/mL), and precultured at 37 °C overnight. The preculture was then seeded at an optical density at 600 nm of 0.1 into the M9 medium containing 100 µM ammonium iron (II) sulfate and 1 mM isopropyl thiogalactoside. The culture was incubated in a 96-deep-well plate at 37 °C for 16 h. The cell culture was sonicated, and 500 µL of the cell lysate was centrifuged to separate the supernatant and pellet fractions, which were used to quantify the amounts of soluble and insoluble forms of AAR and ADO proteins by western blotting. Next, 800 µL of the cell lysate was mixed with an equal amount of ethyl acetate by vortexing. The organic phase was separated from the aqueous phase by centrifugation and was subjected to GC–MS analysis as described elsewhere [14]. Thus, the hydrocarbons both in the *E. coli* cells and in the culture medium are extracted because *E. coli* is known to secrete hydrocarbons into the culture medium [5]. The total amount of hydrocarbons in the *E. coli* cell culture is quantified and reported in this study.

The total amount of hydrocarbons [pentadecane (C15:0), heptadecene (C17:1), and heptadecane (C17:0)] produced in the culture was normalized to the amount of the soluble form of the AAR protein expressed in *E. coli*, which was quantified using western blotting. After normalization, the total amount of hydrocarbons was compared with that of 7942 AAR, and the ratio was used as an index of an aldehyde-producing activity of AAR. The measurements were carried out more than three times, and means ± standard errors are shown.

### Western blotting

Supernatant and pellet samples, prepared from the same amounts of cell lysates, were analyzed by sodium dodecyl sulfate-polyacrylamide gel electrophoresis (SDS-PAGE) using 12 % gels. The proteins were electrotransferred onto a polyvinylidene difluoride membrane (Millipore), and the membrane was blocked with 100 mL of phosphate buffer saline (PBS(–): 8.1 mM Na_2_HPO_4_, 1.47 mM KH_2_PO_4_, 137 mM NaCl, and 2.7 mM KCl) containing 5 % skim milk at room temperature for 1 h with agitation. The membrane was incubated with an anti-His-tag antibody conjugated with horseradish peroxidase (MBL, Japan) at room temperature for 30 min and then rinsed with 100 mL of PBS(–) containing 0.05 % Tween 20 five times. The AAR and ADO proteins were visualized by color reactions with 5 mL of 3,3′,5,5′-tetramethylbenzidine solution (ATTO) at room temperature for ~2 min or for more than 10 min. Gel images of western blotting were acquired using a Gel Doc EZ imager (Bio-Rad). Quantification of the bands of AAR and ADO was performed using the ImageLab software (Bio-Rad).

### Analysis of amino acid sequences

A total of 127 orthologs of AAR were found on the BLAST server [30] using the amino acid sequence of 7942 AAR as a query. Multiple sequence alignment of the 128 AAR amino acid sequences was carried out on the BLAST server. The phylogenetic tree of cyanobacteria based on the AAR amino acid sequences, drawn using NJplot [31], was consistent with that constructed using 16S ribosomal RNA [32]. Multiple sequence alignment of 12 representative AARs was carried out using the Clustal X software [33].

The habitats of 128 cyanobacterial species were examined using MicrobeDB (http://microbedb.jp/), CyanoBase (http://genome.microbedb.jp/cyanobase/) [34], and the Pasteur Culture collection of Cyanobacteria (PCC; http://cyanobacteria.web.pasteur.fr/). Sixty-nine and 52 species are freshwater and marine cyanobacteria, respectively, and seven species can live in both freshwater and marine environments.

## Additional files

**Additional file 1: Table S1.** Cyanobacterial AARs found by the BLAST search. The group number in the phylogenetic tree is shown in the first column. In each group, the cyanobacterial strains are listed in the same order as in Fig. 1. Twelve representative AARs used in the present study are shown in bold.

**Additional file 2: Table S2.** Amino acid sequence identity (%) among the AAR sequences used in the present study.

**Additional file 3: Figure S1.** GC-MS profiles. (a) Control experiments showing that coexpression of AAR and ADO is necessary for hydrocarbon production in* E. coli*. GC-MSprofiles of * E. coli* cell cultures expressing AAR only (red), ADO only (green), and neither AAR nor ADO (black) are shown, but completely overlap. Peaks for pentadecane, heptadecene, and heptadecane, which should appear at 14–17 min, were not observed. (b–d) GC-MS profiles of * E. coli* cell cultures coexpressing AAR from one of eight representative cyanobacteria and 73102 ADO. (b) Whole GC-MS profiles. Pentadecane (C15:0), heptadecene (C17:1), and heptadecane (C17:0) were eluted at the retention time of 14.28, 16.45, and 16.67 min, respectively. (c) Peaks of pentadecane. (d) Peaks of heptadecene and heptadecane. Color codes for various AARs are shown in panel (b).

**Additional file 4: Figure S2.** Characteristics of 7942 AAR determined using * E. coli* strains carrying plasmids with a mutant T7 promoter. In the plasmids, a single-nucleotide mutation as shown in the abscissa is introduced into the T7 promoter region (for example, −17T>A denotes that thymine at the −17th position in the T7 promoter region is replaced by adenine). Strains in which the AAR expression levels are less than 50 % as compared with that of the wild-type T7 promoter are shown in bold. (a) The amounts of pentadecane (C15:0), heptadecene (C17:1), and heptadecane (C17:0) and their combined total amount are shown in red, green, blue, and grey, respectively. The values are normalized to the total amount of hydrocarbons produced in * E. coli* coexpressing 7942 AAR and 73102 ADO. (b, c) The amount of the soluble form (b) and the aldehyde producing activity (c) of 7942 AAR. The values in the ordinate are shown relative to those measured using the * E. coli* strain carrying the wild-type T7 promoter. (d) Fractions of pentadecane, heptadecene, and heptadecane relative to the total amount of hydrocarbons produced in * E. coli*, indicating the substrate specificity of AAR. (e) The amount of the soluble form of 73102ADO.

**Additional file 5: Figure S3.** Characteristics of various AARs determined using the * E. coli* strains carrying plasmids with a −13A>G mutation in the T7 promoter upstream of the AAR gene. (a) The amounts of produced hydrocarbons. (b) The amounts of the soluble form of AARs. (c) The aldehyde producing activity of AARs. (d) Fractions of pentadecane, heptadecene, and heptadecane relative to the total amount of hydrocarbons produced in * E. coli*, indicating the substrate specificity of AAR. The details are the same as in Additional file 4: Figure S2.

**Additional file 6: Figure S4.** Correlation analysis. (a) The activity of AAR plotted against the sequence identity (%) with the amino acid sequence of 7942 AAR. (b–d) The fractions of pentadecane (b), heptadecene (c), and heptadecane (d) plotted against the sequence identity (%) to the amino acid sequence of 9313 AAR,* Ma* AAR, and 73102 AAR, respectively. Because 51142 AAR has a distinct substrate specificity, the data points for 51142 AAR, which are clear outliers in the plots, were omitted. (e, f) The solubility (e) and the expression level (f) of AAR plotted against the sequence identity (%) to the amino acid sequence of 9917 AAR and BP-1 AAR, respectively. In each panel, the red continuous line and blue dotted line indicate the linear regressions obtained using all the data and those obtained using the data without the data point for the highest value. The corresponding correlation coefficients,* r*, and* p* values are shown.

## Abbreviations

0205 AAR: AAR from *Synechococcus* sp. CB0205
1986 AAR: AAR from *Prochlorococcus marinus* subsp. pastoris str. CCMP1986
51142 AAR: AAR from *Cyanothece* sp. ATCC 51142
6803 AAR: AAR from S*ynechocystis* sp. PCC 6803
73102 AAR: AAR from *Nostoc punctiforme* PCC 73102
73102 ADO: ADO from *Nostoc punctiforme* PCC 73102
7336 AAR: AAR from *Synechococcus* sp. PCC 7336
7421 AAR: AAR from *Gloeobacter violaceus* PCC 7421
7942 AAR: AAR from *Synechococcus elongatus* PCC 7942
9313 AAR: AAR from *Prochlorococcus marinus* MIT 9313
9917 AAR: AAR from *Synechococcus* sp. RS9917
AAR: acyl-ACP reductase
ACP: acyl carrier protein
ADO: aldehyde-deformylating oxygenase
BP-1 AAR: AAR from *Thermosynechococcus elongatus* BP-1
GC-MS: gas chromatography-mass spectrometry
LPAAT: lysophosphatidic acid acyltransferase
*Ma* AAR: AAR from *Microcystis aeruginosa*
SDS-PAGE: sodium dodecyl sulfate-polyacrylamide gel electrophoresis
