# Supplementary material for: Comparison of aldehyde-producing activities of cyanobacterial acyl-(acyl carrier protein) reductases
Source: Biotechnol Biofuels. 2016 Nov 1;9:234. doi: 10.1186/s13068-016-0644-5 (PMC5090900; doi:10.1186/s13068-016-0644-5)
Supplement: Supplementary file 1 — Additional file 1: Table S1. Cyanobacterial AARs found by the BLAST search. The group number in the phylogenetic tree is shown in the first column. In each group, the cyanobacterial strains are listed in the same order as in Fig. 1. Twelve representative AARs used in the present study are shown in bold. [file 13068_2016_644_MOESM1_ESM.docx]

**Table S1. Cyanobacterial AARs found by the BLAST search.**

| group  number | Cyanobacterium | accession number | %identity ^a^ |
| --- | --- | --- | --- |
| 1 | *Prochlorothrix hollandica* | WP_017712445 | 69 |
| 1 | *Pseudanabaena biceps* | WP_009628167 | 67 |
| 1 | *Pseudanabaena* sp. PCC 7367 | YP_007104288 | 67 |
| 1 | *Synechococcus* sp. PCC 7502 | YP_007107278 | 66 |
| 1 | *Pseudanabaena* sp. PCC 6802 | WP_019503104 | 71 |
| 1 | ***Thermosynechococcus elongatus* BP-1** | **NP_682102** | **70** |
| 1 | *Synechococcus* sp. PCC 6312 | YP_007061217 | 72 |
| 1 | *Cyanothece* sp. PCC 7425 | WP_012625893 | 70 |
| 1 | *Acaryochloris marina* MBIC11017 | YP_001518341 | 66 |
| 1 | *Acaryochloris* sp. CCMEE 5410 | WP_010474944 | 66 |
| 1 | *Chamaesiphon minutus* PCC 6605 | YP_007099831 | 71 |
| 1 | *Crinalium epipsammum* PCC 9333 | YP_007144708 | 72 |
| 1 | *Coleofasciculus chthonoplastes* | WP_006101798 | 69 |
| 1 | *Microcoleus* sp. PCC 7113 | YP_007123627 | 69 |
| 1 | *Oscillatoriales cyanobacterium* JSC-12 | WP_009768560 | 72 |
| 1 | *Leptolyngbya boryana* | WP_017289932 | 73 |
| 1 | *Nodosilinea nodulosa* | WP_017299924 | 67 |
| 1 | *Chroococcidiopsis thermalis* PCC 7203 | YP_007090946 | 68 |
| 1 | *Geitlerinema* sp. PCC 7407 | YP_007109108 | 70 |
| 1 | *Gloeocapsa* sp. PCC 7428 | YP_007125925 | 72 |
| 1 | *Synechocystis* sp. PCC 7509 | WP_009631650 | 72 |
| 1 | *Calothrix* sp. PCC 6303 | YP_007139249 | 72 |
| 1 | *Calothrix* sp. PCC 7103 | WP_019495251 | 74 |
| 1 | *Rivularia* sp. PCC 7116 | YP_007056781 | 71 |
| 1 | *Richelia intracellularis* | WP_008228511 | 64 |
| 1 | *Richelia intracellularis* | WP_008234504 | 68 |
| 1 | *Mastigocladopsis repens* | WP_017318198 | 71 |
| 1 | *Scytonema hofmanni* | WP_017739657 | 71 |
| 1 | *Fischerella* sp. JSC-11 | WP_009457421 | 72 |
| 1 | *Fischerella muscicola* | WP_016866505 | 73 |
| 1 | *Fischerella muscicola* | WP_016860593 | 73 |
| 1 | *Fischerella* sp. PCC 9339 | WP_017310528 | 73 |
| 1 | *cyanobacterium* PCC 7702 | WP_017322951 | 73 |
| 1 | *Chlorogloeopsis* sp. PCC 9212 | WP_016874619 | 74 |
| 1 | *Cylindrospermopsis raciborskii* | WP_006276489 | 71 |
| 1 | *Raphidiopsis brookii* | WP_009343543 | 71 |
| 1 | *Nostoc azollae* 0708 | YP_003722151 | 72 |
| 1 | *Anabaena* sp. 90 | YP_006995803 | 72 |
| 1 | *Anabaena cylindrica* PCC 7122 | YP_007157703 | 72 |
| 1 | *Anabaena* sp. PCC 7108 | WP_016952716 | 72 |
| 1 | *Nodularia spumigena* | WP_006194203 | 69 |
| 1 | *Microchaete* sp. PCC 7126 | WP_017651282 | 71 |
| 1 | *Calothrix* sp. PCC 7507 | YP_007068745 | 72 |
| 1 | ***Nostoc punctiforme* PCC 73102** | **YP_001865324** | **70** |
| 1 | *Cylindrospermum stagnale* PCC 7417 | YP_007145704 | 72 |
| 1 | *Nostoc* sp. PCC 7120 | NP_489324 | 72 |
| 1 | *Anabaena variabilis* | WP_011319305 | 72 |
| 1 | *Nostoc* sp. PCC 7524 | YP_007077661 | 71 |
| 1 | *Nostoc* sp. PCC 7107 | YP_007048836 | 72 |
| 1 | *Nostoc* sp. KNUA003 | AEV23219 | 72 |
| 1 | *Nostoc* sp. PCC 6720 | AEX28225 | 73 |
| 1 | *Synechococcus* sp. PCC 7335 | WP_006457699 | 67 |
| 1 | *Leptolyngbya* sp. PCC 7375 | WP_006516753 | 68 |
| 1 | *Arthrospira platensis* NIES-39 | YP_005071567 | 68 |
| 1 | *Arthrospira* sp. PCC 8005 | WP_006625675 | 68 |
| 1 | *Lyngbya* sp. PCC 8106 | WP_009783009 | 69 |
| 1 | *Oscillatoria acuminata* PCC 6304 | YP_007085658 | 69 |
| 1 | *Geitlerinema* sp. PCC 7105 | WP_017659190 | 67 |
| 1 | *Oscillatoria* sp. PCC 10802 | WP_017718195 | 71 |
| 1 | *Trichodesmium erythraeum* IMS101 | YP_721978 | 69 |
| 1 | *Oscillatoria nigro-viridis* PCC 7112 | YP_007113927 | 70 |
| 1 | *Microcoleus vaginatus* | WP_006633353 | 71 |
| 1 | *Oscillatoria* sp. PCC 6506 | WP_007353758 | 72 |
| 1 | *Gloeocapsa* sp. PCC 73106 | WP_006530225 | 68 |
| 1 | *Cyanobacterium aponinum* PCC 10605 | YP_007161236 | 69 |
| 1 | *cyanobacterium* UCYN A | YP_003421662 | 65 |
| 1 | *Crocosphaera watsonii* | WP_007306315 | 67 |
| 1 | *Crocosphaera watsonii* | WP_007310823 | 67 |
| 1 | ***Cyanothece* sp. ATCC 51142** | **YP_001802846** | **67** |
| 1 | *Cyanothece* sp. CCY0110 | WP_008274952 | 68 |
| 1 | *Cyanothece* sp. PCC 8801 | YP_002371106 | 69 |
| 1 | *Microcystis aeruginosa* | WP_002800448 | 67 |
| 1 | *Microcystis aeruginosa* | WP_002796085 | 67 |
| 1 | *Microcystis aeruginosa* NIES 843 | YP_001660322 | 67 |
| 1 | *Microcystis aeruginosa* | WP_002755388 | 67 |
| 1 | *Microcystis* sp. T1-4 | WP_008196181 | 67 |
| 1 | *Microcystis aeruginosa* | WP_002787026 | 68 |
| 1 | ***Microcystis aeruginosa*** | **WP_002764636** | **68** |
| 1 | *Microcystis aeruginosa* | WP_002759660 | 67 |
| 1 | *Microcystis aeruginosa* | WP_002780878 | 68 |
| 1 | *Microcystis aeruginosa* | WP_002739243 | 67 |
| 1 | *Microcystis aeruginosa* | WP_002744954 | 67 |
| 1 | *Microcystis aeruginosa* | WP_002778281 | 67 |
| 1 | *Microcystis aeruginosa* | WP_016516573 | 67 |
| 1 | ***Synechocystis* sp. PCC 6803** | **NP_442146** | **68** |
| 1 | *Dactylococcopsis salina* PCC 8305 | YP_007172163 | 63 |
| 1 | *Halothece* sp. PCC 7418 | YP_007167383 | 64 |
| 1 | *Spirulina subsalsa* | WP_017306128 | 70 |
| 2 | *Cyanobium* sp. PCC 7001 | WP_006910260 | 62 |
| 2 | *Cyanobium gracile* PCC 6307 | YP_007044599 | 63 |
| 2 | *Synechococcus* sp. WH 5701 | WP_006171083 | 64 |
| 2 | *Synechococcus* sp. RCC307 | WP_011936002 | 63 |
| 2 | *Synechococcus* sp. CB0101 | WP_010306929 | 61 |
| 2 | ***Synechococcus* sp. CB0205** | **WP_010316605** | **62** |
| 2 | *Prochlorococcus marinus* str. MIT 9211 | YP_001550421 | 61 |
| 2 | *Prochlorococcus marinus* subsp. marinus str. CCMP1375 | NP_874926 | 62 |
| 2 | *Prochlorococcus marinus* str. NATL2A | YP_293055 | 60 |
| 2 | *Prochlorococcus marinus* | WP_011823325 | 61 |
| 2 | *Prochlorococcus marinus* | WP_011376092 | 59 |
| 2 | *Prochlorococcus marinus* str. MIT 9215 | YP_001483815 | 60 |
| 2 | *Prochlorococcus marinus* | WP_002806394 | 60 |
| 2 | *Prochlorococcus marinus* str. AS9601 | YP_001008982 | 60 |
| 2 | *Prochlorococcus marinus* | WP_011862552 | 60 |
| 2 | ***Prochlorococcus marinus* subsp. pastoris str. CCMP1986** | **NP_892651** | **61** |
| 2 | *Prochlorococcus marinus* str. MIT 9515 | YP_001010913 | 61 |
| 2 | ***Prochlorococcus marinus* str. MIT 9313** | **NP_895058** | **63** |
| 2 | ***Synechococcus* sp. RS9917** | **WP_007101949** | **63** |
| 2 | *Synechococcus* sp. RS9916 | WP_007097396 | 64 |
| 2 | *Synechococcus* sp. WH 8016 | WP_006853368 | 57 |
| 2 | *Synechococcus* sp. CC9311 | YP_731192 | 61 |
| 2 | uncultured marine type-A *Synechococcus* GOM 3M9 | ABD96274 | 63 |
| 2 | uncultured marine type-A *Synechococcus* 5B2 | ABB92249 | 63 |
| 2 | uncultured marine type-A *Synechococcus* GOM 5D20 | ABD96480 | 63 |
| 2 | uncultured marine type-A *Synechococcus* GOM 3O12 | ABD96375 | 63 |
| 2 | *Synechococcus* sp. WH 8102 | NP_897828 | 63 |
| 2 | uncultured marine type-A *Synechococcus* GOM 3O6 | ABD96327 | 63 |
| 2 | *Synechococcus* sp. CC9605 | YP_381056 | 64 |
| 2 | *Synechococcus* sp. WH 8109 | WP_006850195 | 64 |
| 2 | *Synechococcus* sp. BL107 | WP_009789282 | 63 |
| 2 | *Synechococcus* sp. CC9902 | WP_011360403 | 63 |
| 2 | *Synechococcus* sp. WH 7805 | WP_006043626 | 63 |
| 2 | *Synechococcus* sp. WH 7803 | WP_011932567 | 63 |
| 2 | *Synechococcus elongatus* PCC 6301 | YP_170761 | 100 |
| 2 | ***Synechococcus elongatus* PCC 7942** | **YP_400611** | **100** |
| 3 | ***Gloeobacter violaceus* PCC 7421** | **NP_926091** | **65** |
| 3 | *Synechococcus* sp. JA-2-3B'a(2-13) | WP_011434004 | 62 |
| 3 | *Synechococcus* sp. JA-3-3Ab | WP_011429322 | 62 |
| 3 | ***Synechococcus* sp. PCC 7336** | **WP_017324926** | **61** |

The group number in the phylogenetic tree is shown in the first column. In each group, the cyanobacterial strains are listed in the same order as in Fig. 1. Twelve representative AARs used in the present study are shown in bold.

^a^ Amino acid sequence identity with the 7942 AAR sequence.
