# Supplementary material for: Comparison of aldehyde-producing activities of cyanobacterial acyl-(acyl carrier protein) reductases
Source: Biotechnol Biofuels. 2016 Nov 1;9:234. doi: 10.1186/s13068-016-0644-5 (PMC5090900; doi:10.1186/s13068-016-0644-5)
Supplement: Supplementary file 2 — Additional file 2: Table S2. Amino acid sequence identity (%) among the AAR sequences used in the present study. [file 13068_2016_644_MOESM2_ESM.docx]

**Table S2. Amino acid sequence identity (%) among the AAR sequences used in the present study.**

|  | PCC 7942 | BP-1 | MIT 9313 | *M. aeruginosa* | PCC 6803 | PCC 7421 | PCC 73102 | PCC 7336 | ATCC 51142 | CCMP 1986 | RS9917 | CB0205 |
| --- | --- | --- | --- | --- | --- | --- | --- | --- | --- | --- | --- | --- |
| PCC 7942 | – | 70 | 63 | 68 | 68 | 65 | 70 | 61 | 67 | 61 | 63 | 62 |
| BP-1 | 70 | – | 59 | 73 | 71 | 67 | 76 | 63 | 72 | 58 | 59 | 58 |
| MIT 9313 | 63 | 59 | – | 57 | 60 | 56 | 62 | 53 | 60 | 73 | 82 | 72 |
| *M. aeruginosa* | 68 | 73 | 57 | – | 76 | 63 | 72 | 63 | 77 | 59 | 59 | 57 |
| PCC 6803 | 68 | 71 | 60 | 76 | – | 62 | 74 | 62 | 78 | 59 | 60 | 59 |
| PCC 7421 | 65 | 67 | 56 | 63 | 62 | – | 67 | 66 | 60 | 55 | 57 | 56 |
| PCC 73102 | 70 | 76 | 62 | 72 | 74 | 67 | – | 66 | 75 | 59 | 62 | 59 |
| PCC 7336 | 61 | 63 | 53 | 63 | 62 | 66 | 66 | – | 60 | 54 | 54 | 53 |
| ATCC 51142 | 67 | 72 | 60 | 77 | 78 | 60 | 75 | 60 | － | 60 | 60 | 58 |
| CCMP1986 | 61 | 58 | 73 | 59 | 59 | 55 | 59 | 54 | 60 | － | 77 | 67 |
| RS9917 | 63 | 59 | 82 | 59 | 60 | 57 | 62 | 54 | 60 | 77 | － | 73 |
| CB0205 | 62 | 58 | 72 | 57 | 59 | 56 | 59 | 53 | 58 | 67 | 73 | － |
