# Supplementary figures and images for: Comparison of aldehyde-producing activities of cyanobacterial acyl-(acyl carrier protein) reductases
Source: Biotechnol Biofuels. 2016 Nov 1;9:234. doi: 10.1186/s13068-016-0644-5 (PMC5090900; doi:10.1186/s13068-016-0644-5)

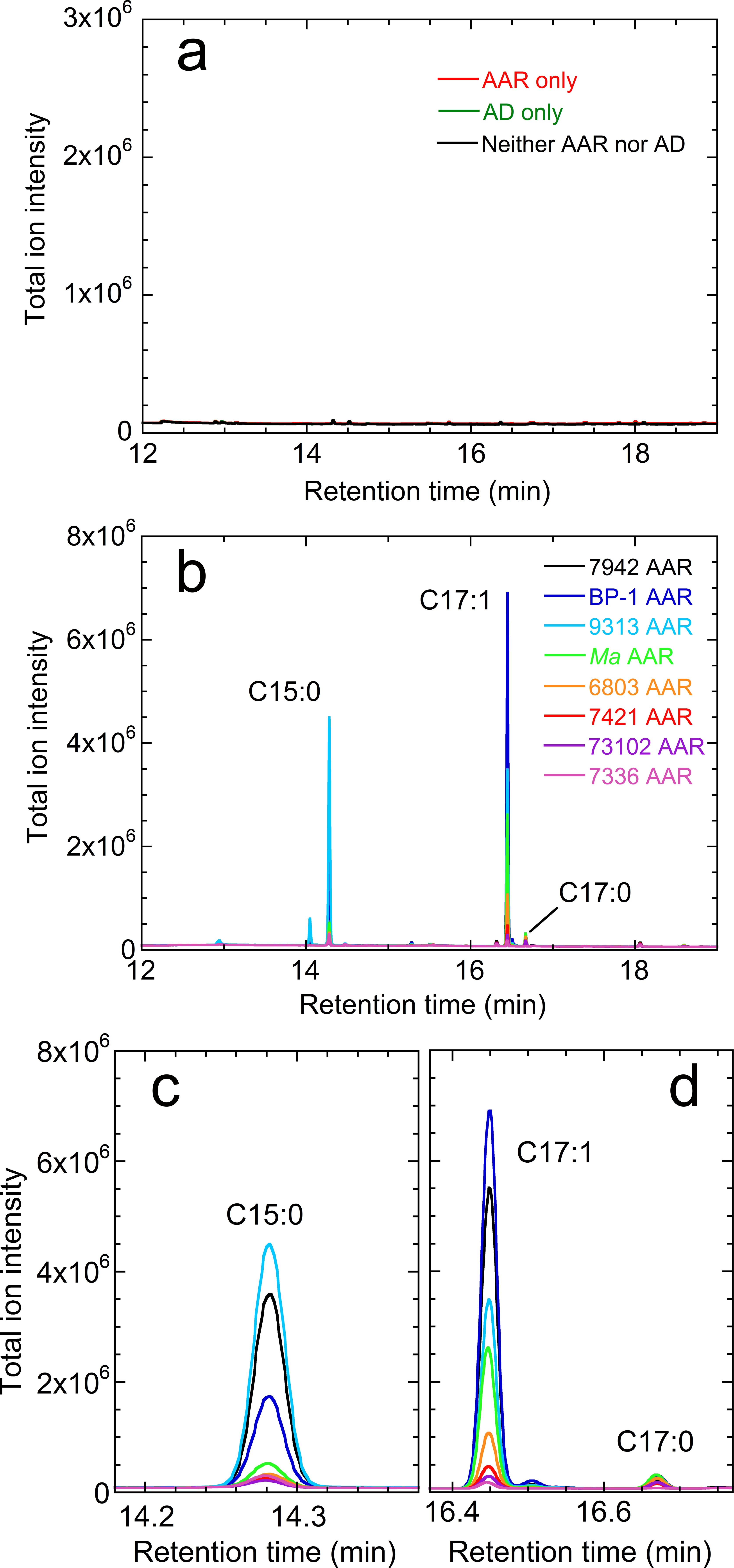

Supplement: Supplementary file 3 — Additional file 3: Figure S1. GC-MS profiles. (a) Control experiments showing that coexpression of AAR and ADO is necessary for hydrocarbon production in E. coli. GC-MSprofiles of E. coli cell cultures expressing AAR only (red), ADO only (green), and neither AAR nor ADO (black) are shown, but completely overlap. Peaks for pentadecane, heptadecene, and heptadecane, which should appear at 14–17 min, were not observed. (b–d) GC-MS profiles of E. coli cell cultures coexpressing AAR from one of eight representative cyanobacteria and 73102 ADO. (b) Whole GC-MS profiles. Pentadecane (C15:0), heptadecene (C17:1), and heptadecane (C17:0) were eluted at the retention time of 14.28, 16.45, and 16.67 min, respectively. (c) Peaks of pentadecane. (d) Peaks of heptadecene and heptadecane. Color codes for various AARs are shown in panel (b). [file 13068_2016_644_MOESM3_ESM.tif]

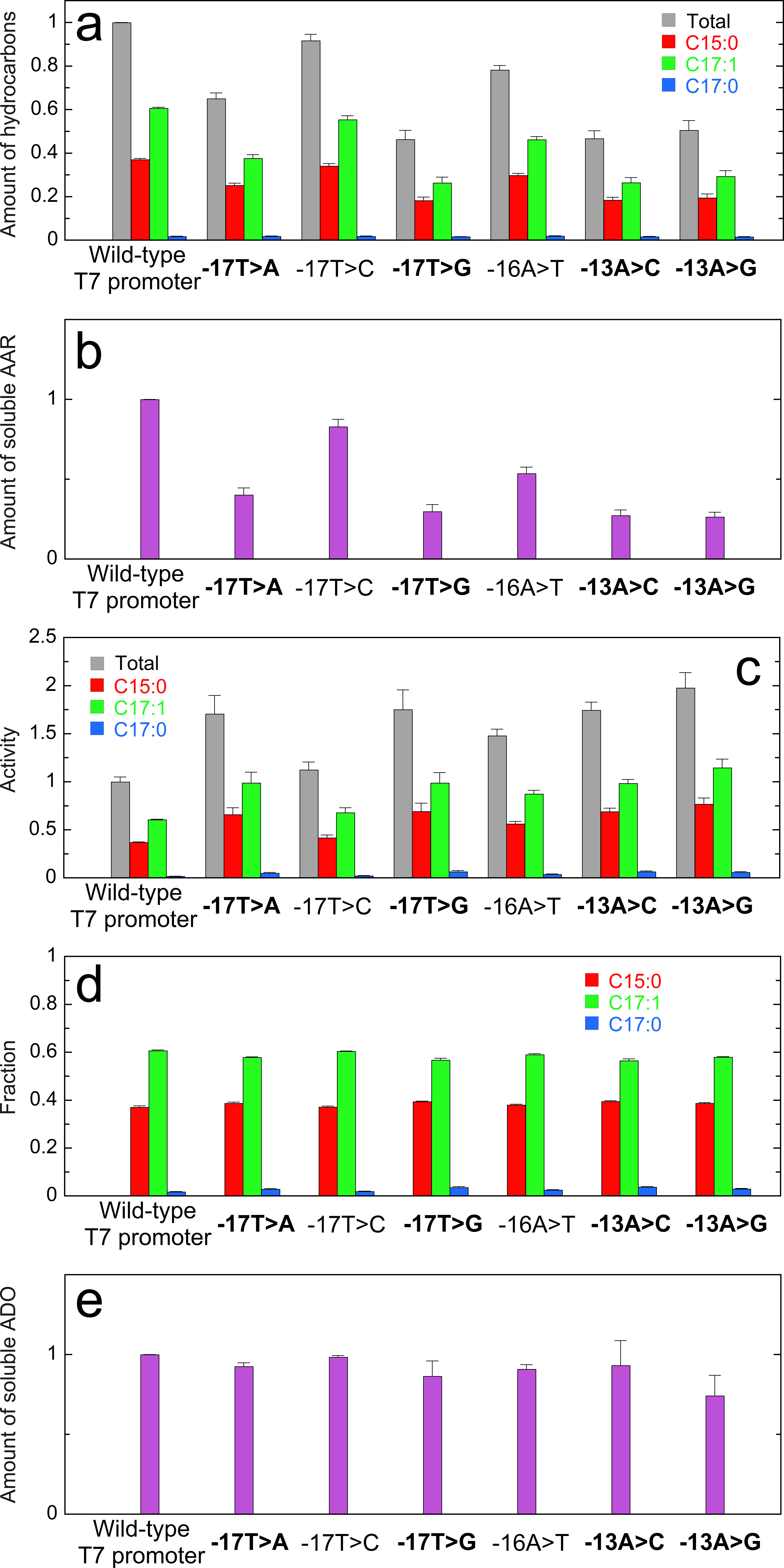

Supplement: Supplementary file 4 — Additional file 4: Figure S2. Characteristics of 7942 AAR determined using E. coli strains carrying plasmids with a mutant T7 promoter. In the plasmids, a single-nucleotide mutation as shown in the abscissa is introduced into the T7 promoter region (for example, −17T>A denotes that thymine at the −17th position in the T7 promoter region is replaced by adenine). Strains in which the AAR expression levels are less than 50 % as compared with that of the wild-type T7 promoter are shown in bold. (a) The amounts of pentadecane (C15:0), heptadecene (C17:1), and heptadecane (C17:0) and their combined total amount are shown in red, green, blue, and grey, respectively. The values are normalized to the total amount of hydrocarbons produced in E. coli coexpressing 7942 AAR and 73102 ADO. (b, c) The amount of the soluble form (b) and the aldehyde producing activity (c) of 7942 AAR. The values in the ordinate are shown relative to those measured using the E. coli strain carrying the wild-type T7 promoter. (d) Fractions of pentadecane, heptadecene, and heptadecane relative to the total amount of hydrocarbons produced in E. coli, indicating the substrate specificity of AAR. (e) The amount of the soluble form of 73102ADO. [file 13068_2016_644_MOESM4_ESM.tif]

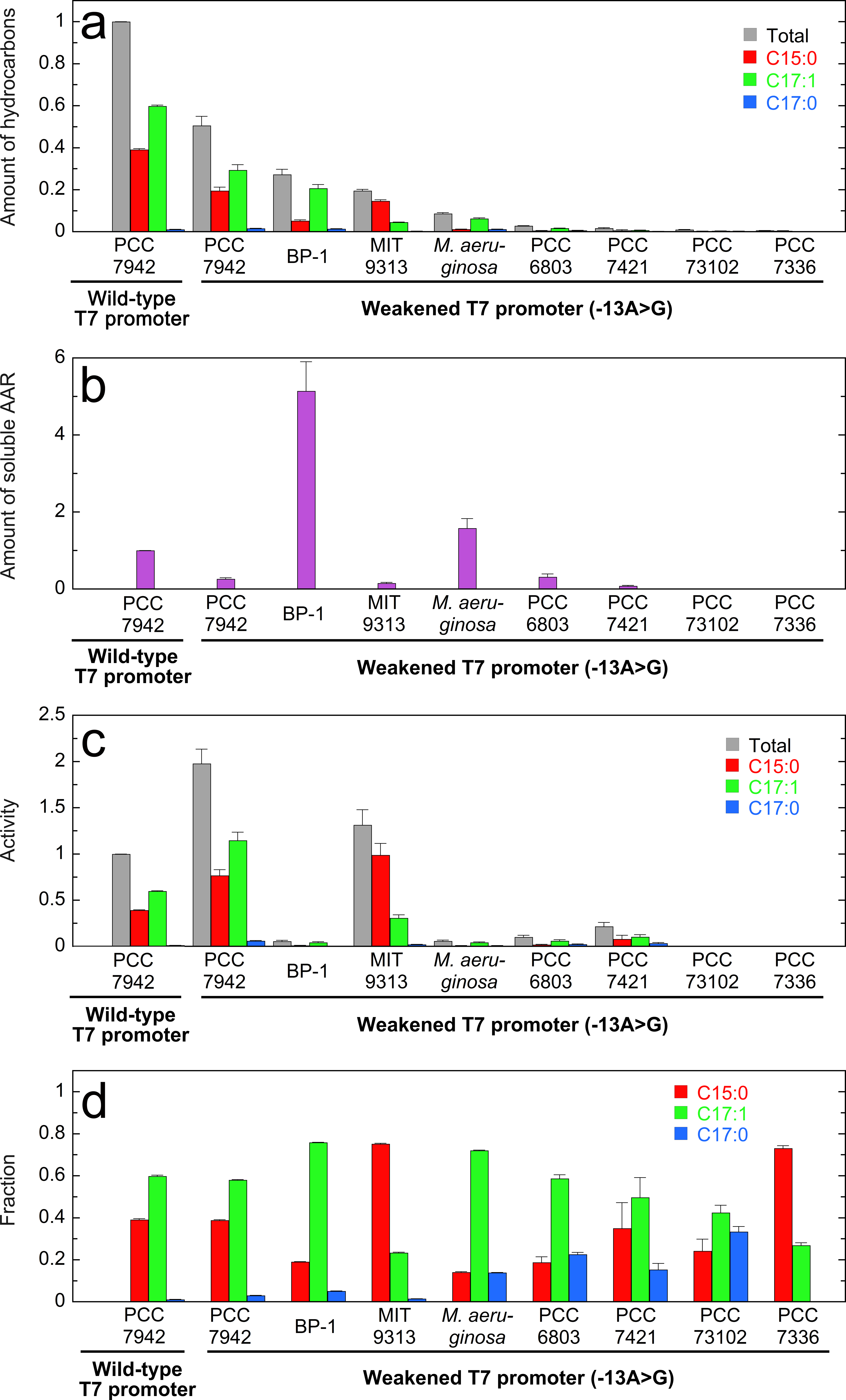

Supplement: Supplementary file 5 — Additional file 5: Figure S3. Characteristics of various AARs determined using the E. coli strains carrying plasmids with a −13A>G mutation in the T7 promoter upstream of the AAR gene. (a) The amounts of produced hydrocarbons. (b) The amounts of the soluble form of AARs. (c) The aldehyde producing activity of AARs. (d) Fractions of pentadecane, heptadecene, and heptadecane relative to the total amount of hydrocarbons produced in E. coli, indicating the substrate specificity of AAR. The details are the same as in Additional file 4: Figure S2. [file 13068_2016_644_MOESM5_ESM.tif]

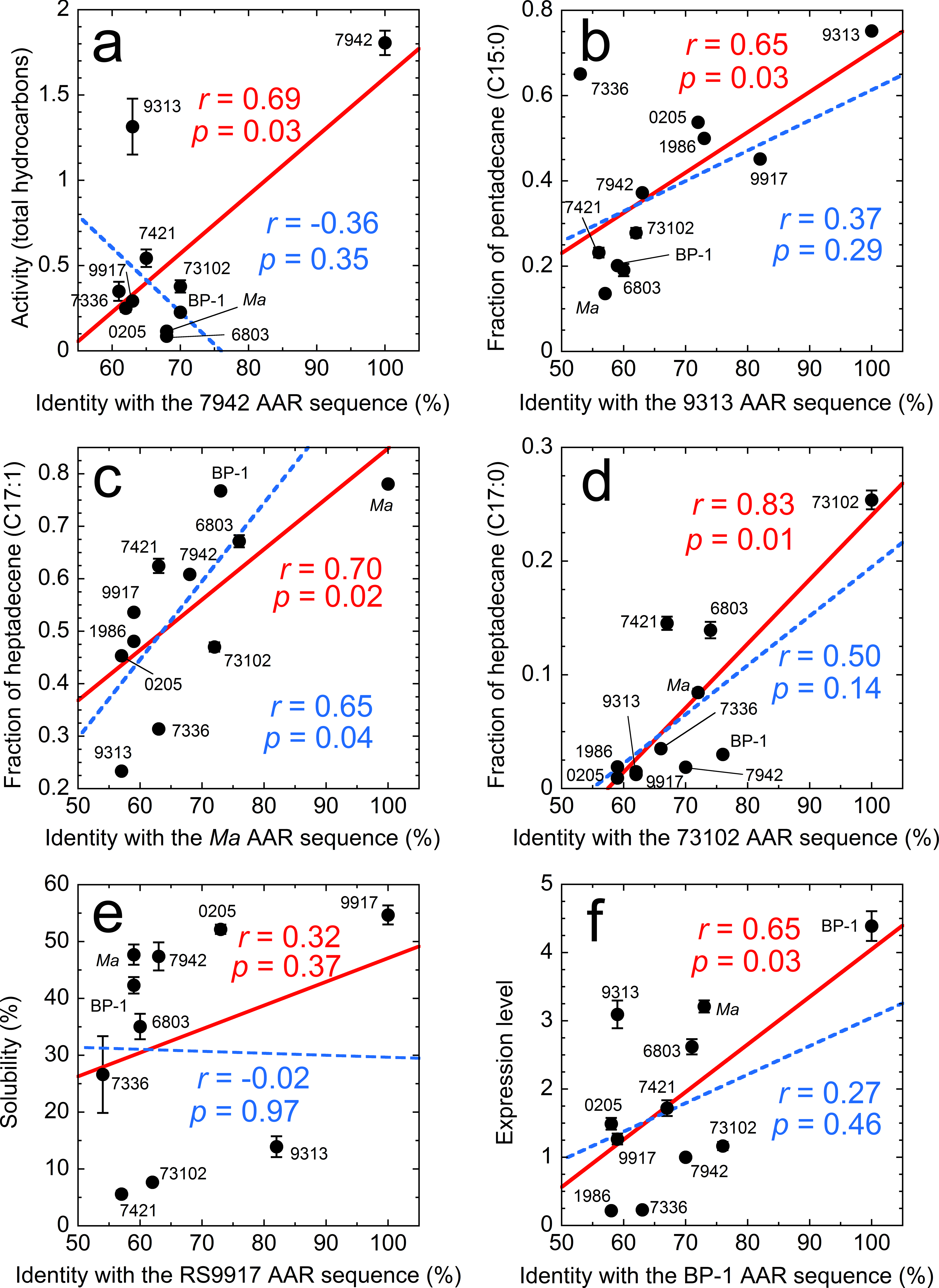

Supplement: Supplementary file 6 — Additional file 6: Figure S4. Correlation analysis. (a) The activity of AAR plotted against the sequence identity (%) with the amino acid sequence of 7942 AAR. (b–d) The fractions of pentadecane (b), heptadecene (c), and heptadecane (d) plotted against the sequence identity (%) to the amino acid sequence of 9313 AAR, Ma AAR, and 73102 AAR, respectively. Because 51142 AAR has a distinct substrate specificity, the data points for 51142 AAR, which are clear outliers in the plots, were omitted. (e, f) The solubility (e) and the expression level (f) of AAR plotted against the sequence identity (%) to the amino acid sequence of 9917 AAR and BP-1 AAR, respectively. In each panel, the red continuous line and blue dotted line indicate the linear regressions obtained using all the data and those obtained using the data without the data point for the highest value. The corresponding correlation coefficients, r, and p values are shown. [file 13068_2016_644_MOESM6_ESM.tif]
